# Golgi dispersal in cancer stem cells promotes chemoresistance of colorectal cancer via the Golgi stress response

**DOI:** 10.1038/s41419-024-06817-0

**Published:** 2024-06-15

**Authors:** Yangkun Li, Lei Mu, Yanqi Li, Yulong Mi, Yibing Hu, Xiaolan Li, Deding Tao, Jichao Qin

**Affiliations:** 1 Molecular Medicine Center, Tongji Hospital, Tongji Medical College, Huazhong University of Science and Technology, Wuhan, 430030 Hubei China; 2 Department of Surgery, Tongji Hospital, Tongji Medical College, Huazhong University of Science and Technology, Wuhan, 430030 Hubei China; 3 Department of Surgical Oncology, Fujian Provincial Hospital, Shengli Clinical Medical College of Fujian Medical University, Fuzhou, 350013 Fujian China; 4 Department of Breast Surgery, Peking University Shenzhen Hospital, Shenzhen, 518000 Guangdong China; 5 Department of Gastrointestinal Surgery, the First Affiliated Hospital, Zhejiang University School of Medicine, Hangzhou, 310003 Zhejiang China

**Keywords:** Cancer stem cells, Cancer therapeutic resistance, Chemotherapy, Golgi, Stress signalling

## Abstract

Chemotherapy is a crucial treatment for colorectal tumors. However, its efficacy is restricted by chemoresistance. Recently, Golgi dispersal has been suggested to be a potential response to chemotherapy, particularly to drugs that induce DNA damage. However, the underlying mechanisms by which Golgi dispersal enhances the capacity to resist DNA-damaging agents remain unclear. Here, we demonstrated that DNA-damaging agents triggered Golgi dispersal in colorectal cancer (CRC), and cancer stem cells (CSCs) possessed a greater degree of Golgi dispersal compared with differentiated cancer cells (non-CSCs). We further revealed that Golgi dispersal conferred resistance against the lethal effects of DNA-damaging agents. Momentously, Golgi dispersal activated the Golgi stress response via the PKCα/GSK3α/TFE3 axis, resulting in enhanced protein and vesicle trafficking, which facilitated drug efflux through ABCG2. Identification of Golgi dispersal indicated an unexpected pathway regulating chemoresistance in CRC.

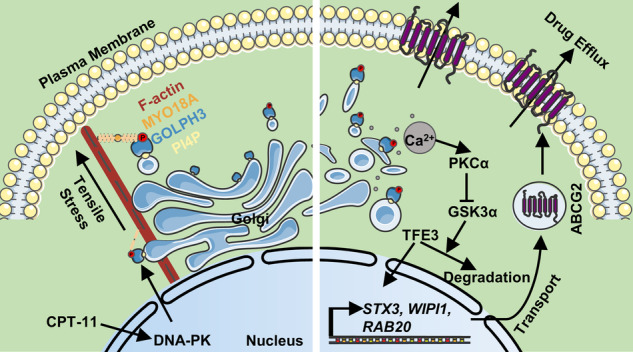

## Introduction

Colorectal cancer (CRC) ranks as the third-leading cause of cancer incidence and mortality worldwide [1]. The mortality rate continues to increase in younger adults [2]. Chemotherapy is the major treatment for postoperative, advanced, or metastatic CRCs, which minimizes the risk of postoperative recurrence and prolongs survival [3]. However, the development of chemoresistance severely inhibits the efficiency of chemotherapy and results in therapeutic failure [4]. Chemoresistance often occurs due to the presence of cancer stem cells (CSCs), which may be intrinsically resistant to therapy or extrinsically induced by the tumor microenvironment to acquire resistance [5]. Therefore, it is crucial to investigate the specific mechanisms underlying chemoresistance in CSCs to develop more effective therapies for CRCs.

Golgi dispersal is a cytoplasmic response to DNA damage, resulting in a dramatic change in Golgi morphology from the perinuclear ribbon to punctate fragments dispersed throughout the cytoplasm [6]. Golgi membranes are linked by golgi phosphoprotein 3 (GOLPH3) to the unconventional myosin MYO18A and F-actin, which exert tensile stress to stretch the Golgi around the nucleus and promote transport [7, 8]. DNA damage induced by chemotherapeutic agents, such as camptothecin and doxorubicin, leads to the phosphorylation of GOLPH3 via DNA-PK, which triggers Golgi dispersal [6, 9]. Interestingly, *GOLPH3* has been identified as an oncogene that is amplified in human malignancies and is overexpressed in human urothelial bladder CSCs [10, 11]. Interference with Golgi dispersal by depletion of GOLPH3 reduces the resistance to DNA-damaging agents in HeLa cells [6, 8]. However, Golgi dispersal in colorectal CSCs and the mechanisms by which it regulates chemoresistance in CRCs remain unclear.

Golgi apparatus is responsible for the post-translational modification and transport of proteins and vesicles [12–14]. Golgi stress response is triggered when the cellular demand exceeds the capacity of Golgi [15]. Golgi stress response, which induces Golgi disassembly or fragmentation, has been reported in several diseases, including viral infections, neurodegenerative disorders, and liver diseases [16, 17]. Golgi stress response prevents apoptosis via the transcription factor E3 (TFE3) pathway [18]. TFE3 is dephosphorylated and translocated to the nucleus, where it activates the transcription of TFE3-target genes, including glycosylation enzymes and vesicular transport proteins [19]. Notably, Golgi dispersal induced by DNA-damaging agents is morphologically similar to the changes in Golgi that occur as a result of Golgi stress response. However, it is unknown whether Golgi dispersal induced by DNA-damaging agents is a form of Golgi stress response.

Here, we demonstrated that DNA damage caused by chemotherapeutic agents triggered the dispersal of Golgi throughout the cytoplasm in CRC cells. In addition, colorectal CSCs displayed a greater degree of Golgi dispersal than non-CSCs, which was dependent on GOLPH3. Moreover, Golgi dispersal was required for CRC cell survival after treatment with DNA-damaging agents. Furthermore, Golgi dispersal induced Golgi stress response via the TFE3 pathway and enhanced protein and vesicle trafficking. ABCG2 was enhanced by Golgi stress response and promoted drug efflux. Thus, we identified a distinctive form of Golgi stress response with profound implications for the cellular response to chemotherapeutic agents.

## Results

### DNA-damaging agents induce Golgi dispersal in CRC cells

**Fig. 1:**
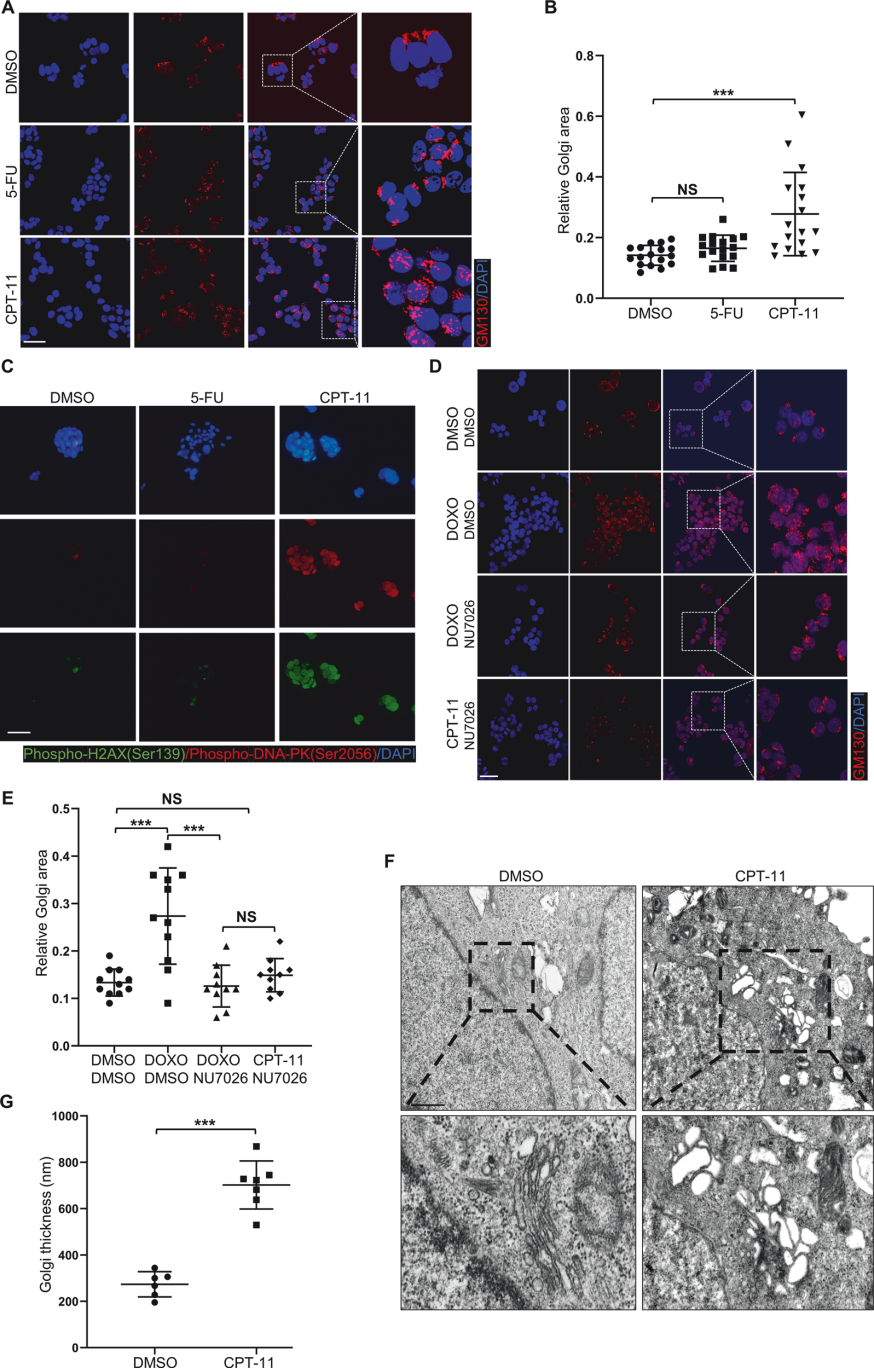
DNA-damaging agents induce Golgi dispersal in CRC cells. **A** XhCRC cells treated with DMSO (control), 5-FU (1 μM), or CPT-11 (1 μM) for 24 h were stained with GM130 (cis-Golgi) and DAPI (nucleus). Scale bar: 20 μm. **B** Relative Golgi area per cell was measured in DMSO-, 5-FU-, or CPT-11-treated XhCRC cells. The data are shown as the mean ± SD (*n* = 17). ^***^P < 0.001. NS no significance. **C** XhCRC cells treated with DMSO (control), 5-FU (1 μM), or CPT-11 (1 μM) for 24 h were stained with indicated antibodies and DAPI (nucleus). Scale bar: 20 μm. **D** XhCRC cells treated with DMSO (control), DOXO (20 nM), or CPT-11 (1 μM) alone or with NU7026 (10 μM) for 24 h were stained with GM130 (cis-Golgi) and DAPI (nucleus). Scale bar: 20 μm. **E** Relative Golgi area per cell was measured in DMSO-, DOXO-, or CPT-11-treated XhCRC cells. The data are shown as the mean ± SD (*n* = 11). ^***^P < 0.001. NS no significance. **F** Representative TEM images of the XhCRC cells treated with DMSO (control) or CPT-11 (1 μM) for 24 h. Scale bar: 1 μm. **G** Golgi thickness per cell was measured in DMSO- or CPT-11-treated XhCRC cells. The data are shown as the mean ± SD (*n* = 7). ^***^P < 0.001.

To investigate the relationship between chemotherapy and Golgi dispersal in CRC cells, the XhCRC and SW620 cell lines were treated with 5-fluorouracil (5-FU) or irinotecan (CPT-11). 5-FU affects pyrimidine synthesis by inhibiting thymidylate synthetase [20], whereas CPT-11 is a DNA topoisomerase I inhibitor that results in double-strand breaks and DNA-PK activation [21]. Exposure to CPT-11, but not 5-FU, caused Golgi morphology to change from the perinuclear ribbon to punctate fragments dispersed throughout the cytoplasm (Fig. 1A). Quantitative measurement of the Golgi area per cell indicated that Golgi in CPT-11-treated cells was significantly greater than that in the control or 5-FU-treated cells (Fig. 1B). Golgi dispersal has been found to be dependent on DNA-PK [6]. Using DNA-PK related markers such as phospho-H2AX (Ser139) and phospho-DNA-PK (Ser2056) [22], we identified considerable DNA-PK activation in the CPT-11-treated group (Fig. 1C). To further confirm the requirement of DNA-PK in Golgi dispersal, XhCRC cells were also treated with doxorubicin (DOXO), which inhibits DNA topoisomerase I/II and activates DNA-PK [23], as well as the DNA-PK inhibitor NU7026 [24]. Golgi dispersal was also induced by DOXO, and both DOXO- and CPT-11-induced Golgi dispersal were inhibited by NU7026 (Fig. 1D, E). Golgi morphology was further assessed via transmission electron microscopy. Consistently, Golgi apparatus of CPT-11-treated CRC cells exhibited a loose state and vacuole-like alterations, with a significant increase in Golgi thickness, in contrast to the typical stacked structure observed in control cells (Fig. 1F, G). Taken together, these findings indicate that Golgi dispersal in CRC cells is induced by DNA-damaging agents.

### CPT-11-induced Golgi dispersal is independent of apoptosis

**Fig. 2:**
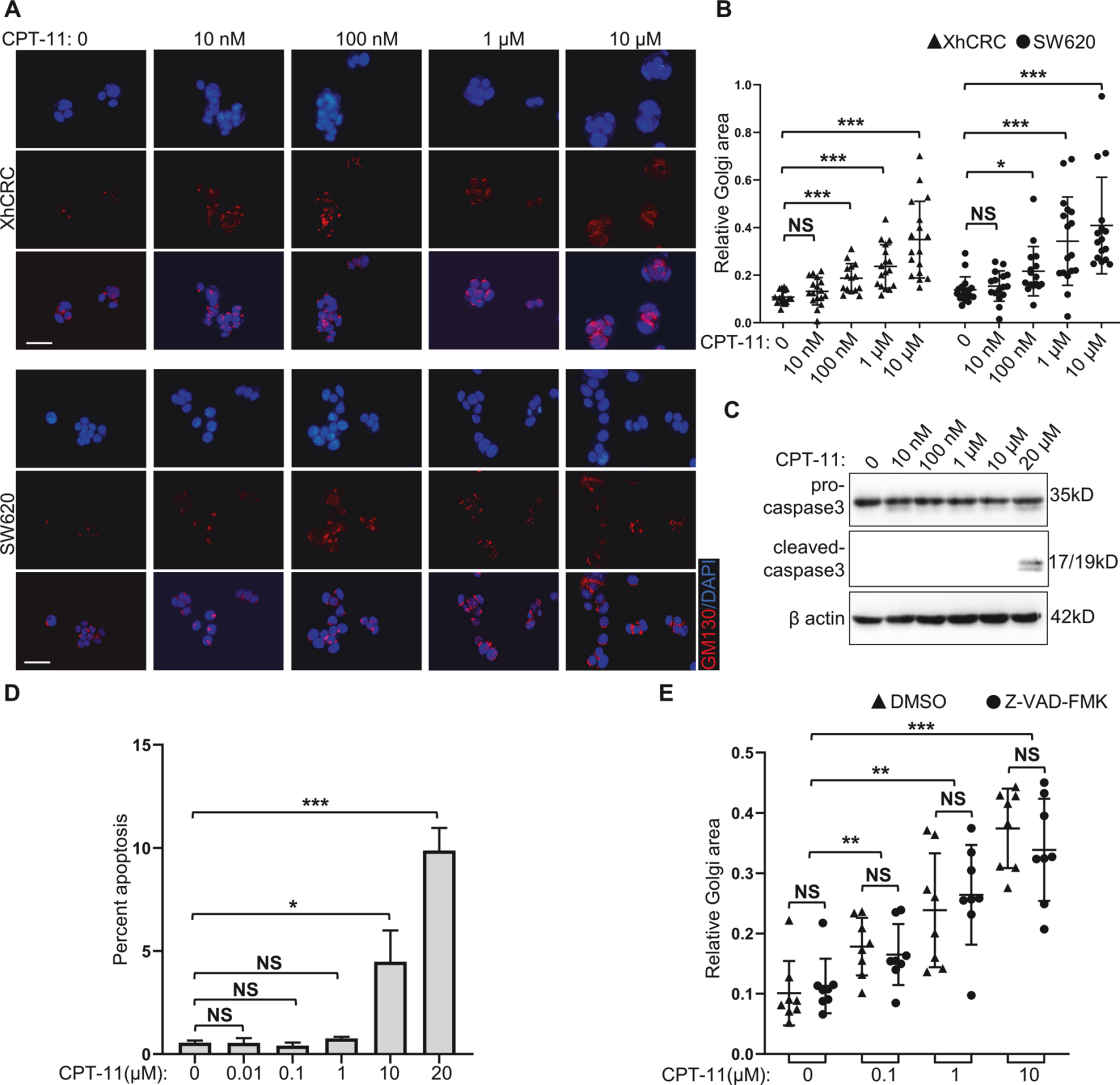
CPT-11-induced Golgi dispersal is independent of apoptosis. **A** XhCRC and SW620 cells treated with the indicated doses of CPT-11 for 24 h were stained with GM130 (cis-Golgi) and DAPI (nucleus). Scale bar: 20 μm. **B** Relative Golgi area per cell was measured in CPT-11-treated XhCRC and SW620 cells. The data are shown as the mean ± SD (*n* = 17). ^*^P < 0.05, ^***^P < 0.001. NS no significance. **C** Western blot analysis of pro-caspase 3 and cleaved-caspase 3 in XhCRC cells treated with the indicated doses of CPT-11 for 24 h. **D** XhCRC cells were treated with the indicated doses of CPT-11 for 24 h. Graphed are the percentage of apoptotic cells. The data are shown as the mean ± SD (*n* = 3). ^*^P < 0.05, ^***^P < 0.001. NS no significance. **E** XhCRC cells were treated with the indicated doses of CPT-11 for 24 h, but also pretreated with DMSO (control) or Z-VAD-FMK (40 μM) for 30 min. Relative Golgi area per cell was measured. The data are shown as the mean ± SD (*n* = 8). ^**^P < 0.01, ^***^P < 0.001. NS no significance.

During apoptosis, Golgi apparatus is fragmented by activated caspases, which is morphologically similar to CPT-11-induced Golgi dispersal [25, 26]. We investigated whether CPT-11-induced Golgi dispersal is a result of apoptosis. XhCRC and SW620 cells displayed significant Golgi dispersal when treated with 100 nM CPT-11, and the degree of Golgi dispersal increased as the dose of CPT-11 increased (Fig. 2A, B). Cleaved caspase-3 and annexin V were used to detect apoptosis [27, 28]. Treatment with modest doses of CPT-11 (0.01–1 μM) caused no significant apoptosis in XhCRC cells (Fig. 2C, D, and Supplementary Fig. S1A), consistent with other morphological evidence of apoptosis, such as pyknotic or fragmented nuclei (Fig. 2A). The nonapoptotic cells, which comprised most of the cells, exhibited a uniformly dispersed Golgi (Fig. 2A). To further eliminate the impact of apoptosis on Golgi apparatus, XhCRC cells were pretreated with Z-VAD-FMK, a pan-caspase inhibitor [29]. The addition of Z-VAD-FMK did not alter the degree of Golgi dispersal in XhCRC cells treated with CPT-11 (0.1–10 μM) (Fig. 2E and Supplementary Fig. S1B). These results demonstrate that CPT-11-induced Golgi dispersal occurs independently of apoptosis.

### Golgi dispersal is highly induced in colorectal CSCs

**Fig. 3:**
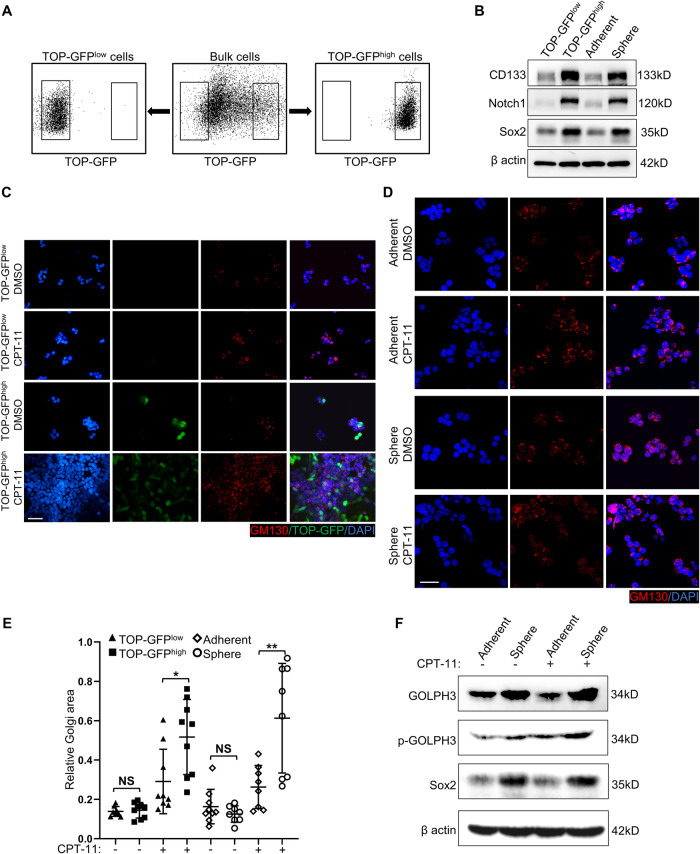
Golgi dispersal is highly induced in colorectal CSCs. **A** Post-sorting analysis of the sorted TOP-GFP^low^ and TOP-GFP^high^ XhCRC cells. **B** Western blot analysis of the indicated molecules in TOP-GFP^low^, TOP-GFP^high^, adherent (monolayer-cultured cells), and sphere (sphere-derived cells) XhCRC cells. **C** TOP-GFP^low^ and TOP-GFP^high^ XhCRC cells treated with DMSO (control) or CPT-11 (1 μM) for 24 h were stained with GM130 (cis-Golgi) and DAPI (nucleus). Scale bar: 20 μm. **D** Adherent and sphere XhCRC cells treated with DMSO (control) or CPT-11 (1 μM) for 24 h were stained with GM130 (cis-Golgi) and DAPI (nucleus). Scale bar: 20 μm. **E** Relative Golgi area per cell was measured in DMSO- or CPT-11-treated TOP-GFP^low^, TOP-GFP^high^, adherent, and sphere XhCRC cells. The data are shown as the mean ± SD (*n* = 9). ^*^P < 0.05, ^**^P < 0.01. NS no significance. **F** Western blot analysis of GOLPH3 and p-GOLPH3 (phosphorylated GOLPH3) in adherent and sphere XhCRC cells treated with DMSO (control) or CPT-11 (1 μM) for 24 h.

To investigate whether there are heterogeneous dispersals of Golgi, we investigated Golgi dispersal in colorectal CSCs and non-CSCs. XhCRC and SW620 cells were transfected with a TOP-GFP reporter that labels tumor cells with high intrinsic Wnt activity, which is a distinguishing marker of CSCs [30–32]. TOP-GFP^low^ and TOP-GFP^high^ cells were sorted by fluorescence-activated cell sorting (FACS) (Fig. 3A). CSCs can also be enriched in tumor spheres under a serum-free, non-adhesive environment, whereas non-CSCs are enriched in monolayer culture [33, 34]. Colorectal tumorsphere formation (sphere-derived cells) and monolayer cell culture (adherent cells) were used in our study (Supplementary Fig. S2A). Compared with TOP-GFP^low^ cells or adherent cells, TOP-GFP^high^ cells or sphere-derived cells exhibited increased expression of stemness-associated markers (i.e., CD133, Notch1, Sox2, and Wnt) (Fig. 3B and Supplementary Fig. S2B). Notably, neither TOP-GFP^low^ cells or TOP-GFP^high^ cells, adherent cells or sphere-derived cells exhibited discernible differences in Golgi morphology (Fig. 3C-E). After treatment with CPT-11, TOP-GFP^high^ or sphere-derived cells displayed a greater degree of Golgi dispersal compared with TOP-GFP^low^ or adherent cells (Fig. 3C–E). Phosphorylated GOLPH3 is required for Golgi dispersal [6, 8]. After treatment with CPT-11, the phosphorylated GOLPH3 levels were higher in sphere-derived CSCs than in adherent non-CSCs (Fig. 3F). These findings indicate that, following CPT-11 treatment, CSCs exhibit a greater Golgi dispersal compared with non-CSCs in CRCs.

### Golgi dispersal confers resistance to CPT-11 in colorectal CSCs

**Fig. 4:**
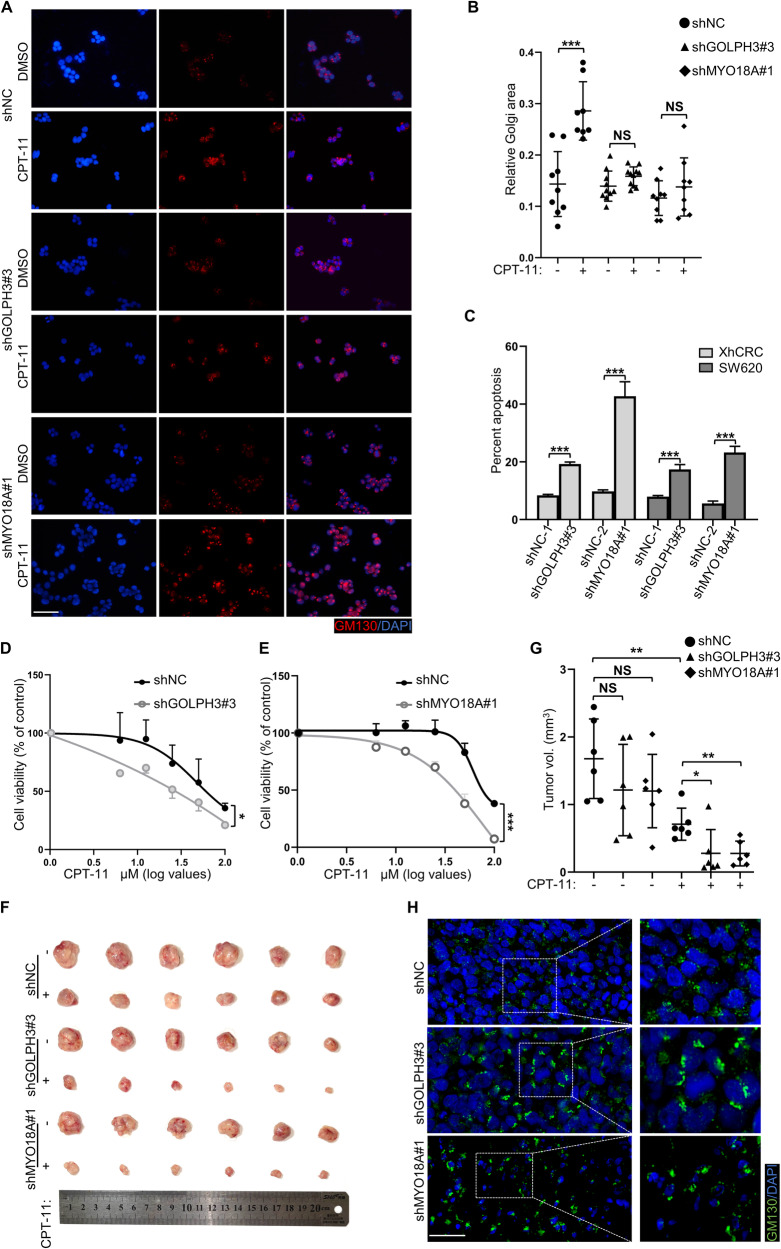
Golgi dispersal confers resistance to CPT-11 in colorectal CSCs. **A** ShNC, shGOLPH3, and shMYO18A sphere-derived XhCRC cells treated with DMSO (control) or CPT-11 (1 μM) for 24 h were stained with GM130 (cis-Golgi) and DAPI (nucleus). Scale bar: 20 μm. **B** Relative Golgi area per cell was measured. The data are shown as the mean ± SD (*n* = 11). ^***^P < 0.001. NS no significance. **C** ShNC, shGOLPH3, and shMYO18A sphere-derived XhCRC or SW620 cells were treated with CPT-11 (20 μM) for 24 h. Graphed are the percentage of apoptotic cells. The data are shown as the mean ± SD (*n* = 3). ^***^P < 0.001. **D**, **E** Cell viability was measured relative to control in shNC, shGOLPH3, and shMYO18A sphere-derived XhCRC cells treated with indicated doses of CPT-11 for 24 h. The data are shown as the mean ± SD (*n* = 3). ^*^P < 0.05, ^***^P < 0.001. **F** The images of tumors from shNC, shGOLPH3, and shMYO18A groups treated with DMSO (control) or CPT-11 (100 mg/kg). **G** Indicated tumor volume was measured. The data are shown as the mean ± SD (*n* = 6). ^*^P < 0.05, ^**^P < 0.01. NS no significance. **H** ShNC, shGOLPH3, and shMYO18A xenografts treated with CPT-11 were stained with GM130 (cis-Golgi) and DAPI (nucleus). Scale bar: 20 μm.

Golgi dispersal is regulated by the GOLPH3/MYO18A/F-actin pathway [8, 35]. To further determine the role of GOLPH3 or MYO18A in Golgi dispersal, GOLPH3 and MYO18A were knocked down by shRNAs (Supplementary Fig. S3A, B). Notably, knockdown of either GOLPH3 or MYO18A resulted in considerable inhibition of CPT-11-induced Golgi dispersal in sphere-derived XhCRC cells (Fig. 4A, B). Following knockdown of either GOLPH3 or MYO18A, the percentage of apoptosis in sphere-derived XhCRC or SW620 cells significantly increased (Fig. 4C and Supplementary Fig. S3C, D). In addition, the viability of sphere-derived XhCRC or SW620 cells was decreased by GOLPH3 or MYO18A knockdown (Fig. 4D, E, and Supplementary Fig. S3E, F). ShNC, shGOLPH3, and shMYO18A sphere-derived XhCRC cells were injected subcutaneously into NOD/Scid mice. After treatment with CPT-11, the tumor volumes were significantly decreased in the shGOLPH3 and shMYO18A groups than in the shNC group (Fig. 4F, G). Interestingly, the Golgi structure was looser in CPT-11-treated shNC cell-derived xenografts than in shGOLPH3 or shMYO18A groups (Fig. 4H). In conclusion, Golgi dispersal is required for the survival of colorectal CSCs following CPT-11 treatment.

### Golgi dispersal triggers Golgi stress response via the TFE3 pathway

**Fig. 5:**
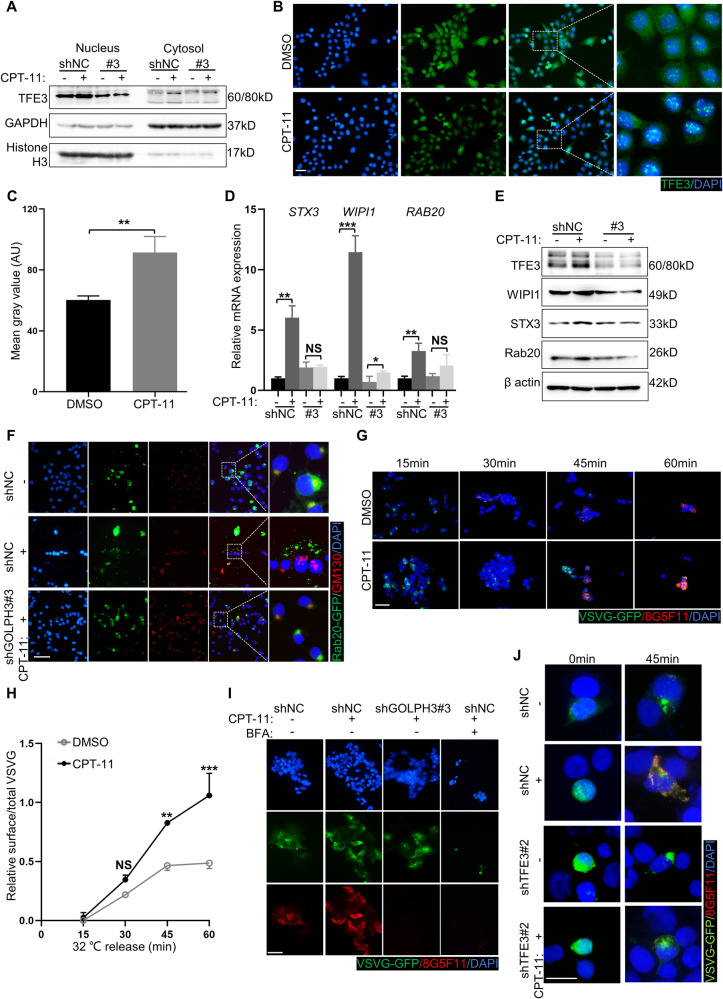
Golgi dispersal triggers the Golgi stress response via the TFE3 pathway. **A** Western blot analysis of TFE3 in the nucleus and cytosol of XhCRC cells treated with DMSO (control) or CPT-11 (1 μM) for 24 h. **B** XhCRC cells treated with DMSO (control) or CPT-11 (1 μM) for 24 h were stained with TFE3 and DAPI (nucleus). Scale bar: 20 μm. **C** The mean gray value of TFE3 was measured. The data are shown as the mean ± SD (*n* = 3). ^**^P < 0.01. **D**, **E** Expression levels of indicated mRNAs and proteins in shNC and shGOLPH3 (#3) XhCRC cells treated with DMSO (control) or CPT-11 (1 μM) for 24 h. The data are shown as the mean ± SD (*n* = 3). ^*^P < 0.05, ^**^P < 0.01, ^***^P < 0.001. NS no significance. **F** ShNC and shGOLPH3 XhCRC cells treated with DMSO (control) or CPT-11 (1 μM) for 24 h were stained with Rab20-GFP, GM130 (cis-Golgi), and DAPI (nucleus). Scale bar: 20 μm. **G** XhCRC cells treated with DMSO (control) or CPT-11 (1 μM) for 24 h were stained with VSVG-GFP, 8G5F11, and DAPI (nucleus) at different 32 °C release times. Scale bar: 20 μm. **H** Relative surface/total VSVG was measured. The data are shown as the mean ± SD (*n* = 3). ^**^P < 0.01, ^***^P < 0.001. NS no significance. **I** ShNC and shGOLPH3 XhCRC cells were treated with DMSO (control) or CPT-11 (1 μM) for 24 h alone or with BFA (1 μM) for 1 h. Cells were stained with VSVG-GFP, 8G5F11, and DAPI (nucleus) at 45 min. Scale bar: 20 μm. **J** ShNC and shTFE3 XhCRC cells treated with DMSO (control) or CPT-11 (1 μM) for 24 h were stained with VSVG-GFP, 8G5F11, and DAPI (nucleus) at different 32 °C release times. Scale bar: 10 μm.

Golgi dispersal induced by CPT-11 mimics Golgi disassembly or fragmentation in the Golgi stress response, which activates the TFE3 pathway to regulate Golgi function [18, 36, 37]. GOLPH3 has also been identified as a potential stress receptor for Golgi [38]. Thus, we inferred that CPT-11-induced Golgi dispersal triggers Golgi stress response via the TFE3. Compared with the control group, total (nuclear and cytoplasmic) and nuclear TFE3 levels were increased in CPT-11-treated XhCRC cells (Fig. 5A–C), whereas this trend was reversed by GOLPH3 knockdown (Fig. 5A). TFE3 activates the transcription of Golgi-associated genes, including those involved in Golgi enzymes (*SIAT4A* and *FUT1*), Golgi structural proteins (*GM130* and *Giantin*), and vesicular transport components (*STX3*, *WIPI1*, and *RAB20*) [39–41]. Compared with control cells, CPT-11-treated cells displayed elevated expression of Golgi-associated genes, which was reduced in the shGOLPH3 group (Fig. 5D, E and Supplementary Fig. S4A, B). To further investigate the association between Golgi dispersal and Golgi stress response, XhCRC cells were treated with Golgi stressor monensin [37, 39]. Monensin caused Golgi dispersal and increased levels of TFE3 and its downstream proteins (STX3, WIPI1, and Rab20) (Supplementary Fig. S4C, D). We also assessed whether ER or lysosome undergo stress response in CPT-11-treated CRC cells. ER stress was not induced by CPT-11 in LoVo and SW620 cells like it was by ER stressor tunicamycin [42], which was verified by measuring ER-stress mRNAs (spliced *XBP1*, *BIP*, and *CHOP*) (Supplementary Fig. S4E, F). In addition, lysosome stress was caused by starvation [43] rather than CPT-11, which was verified by measuring lysosomal proteins (Lamp1 and Cathepsin D) and nuclear localization of TFEB (Supplementary Fig. S4G–I). Thus, CPT-11-induced Golgi dispersal is a specific form of Golgi stress response.

The Golgi stress response regulates Golgi functions, including protein glycosylation and protein and vesicle transport [18]. Protein glycosylation was verified by fluorescently labeled wheat germ agglutinin (WGA) lectin, which selectively binds to N-acetylneuraminic acid (sialic acid) and N-acetylglucosamine (GlcNAc) on the PM [44], and individual glycoprotein Lamp2, whose mobility on gels was impacted by glycosylation [45]. Compared with control cells, the cell surface signal for WGA was not affected in CPT-11-treated cells (Supplementary Fig. S5A–C). As the dose of CPT-11 increased, Lamp2 mobility did not change (Supplementary Fig. S5D). These results reveal that CPT-11-induced Golgi dispersal does not impair glycosylation. Vesicle and protein transport were verified by tracking the transport of Rab20-GFP, which is a marker of Golgi-derived vesicles [46, 47], and temperature-sensitive mutant (ts045)-vesicular stomatitis virus glycoprotein (VSVG)-GFP, which is a representative protein transported from Golgi to the PM [48]. Rab20-GFP appeared as dense clumps and co-localized with Golgi in the perinuclear region in the control group, while punctate Rab20 was dispersed throughout the cytoplasm in CPT-11-treated cells, indicating accelerated vesicle trafficking (Fig. 5F). Furthermore, this response was inhibited by GOLPH3 knockdown (Fig. 5F). To quantitatively measure the trafficking rate of VSVG-GFP, we labeled the VSVG on cell surface with an 8G5F11 antibody against the extracellular domain of VSVG and calculated the ratio of surface VSVG to total VSVG-GFP signal. Notably, VSVG-GFP was transported much faster in CPT-11-treated cells compared with the control group, resulting in an increase in the ratio of surface-to-total VSVG (Fig. 5G, H). Knockdown of GOLPH3 significantly reduced the transport of VSVG-GFP like protein transport inhibitor brefeldin A (BFA) [49, 50] (Fig. 5I). Furthermore, Golgi-stress protein TFE3 was knocked down (Supplementary Fig. S5E). Knockdown of TFE3 restricted the transport of VSVG-GFP (Fig. 5J). Taken together, Golgi dispersal triggers the Golgi stress response and enhances both vesicle and protein transport via the TFE3 pathway.

### The function of TFE3 is mediated via the PKCα/GSK3α pathway

**Fig. 6:**
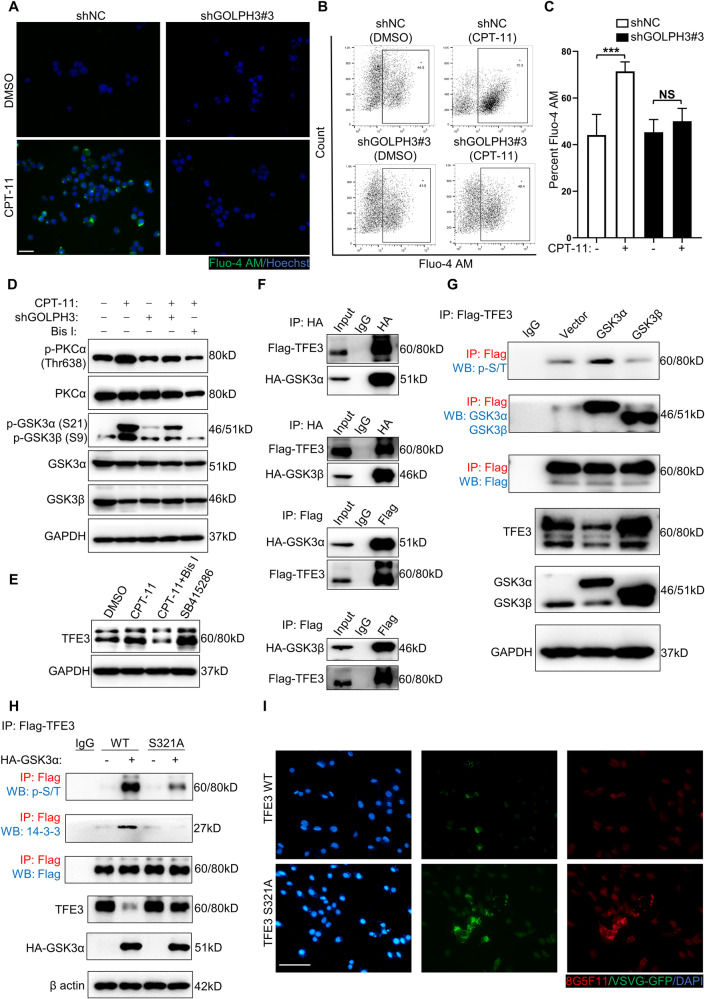
TFE3 is mediated via the PKCα/GSK3α pathway. **A** ShNC and shGOLPH3 XhCRC cells treated with DMSO (control) or CPT-11 (1 μM) for 24 h were stained with Fluo-4 AM (Ca^2+^) and Hoechst (nucleus). Scale bar: 20 μm. Graphed are the flow cytometric analysis of Fluo-4 AM (**B**) and the percentage of Fluo-4 AM-positive cells (**C**). The data are shown as the mean ± SD (*n* = 6). ^***^P < 0.001. NS no significance. **D** Western blot analysis of the indicated molecules in shNC and shGOLPH3 XhCRC cells treated with DMSO (control) or CPT-11 (1 μM) for 24 h alone or with Bis I (20 nM) for 1 h. **E** Western blot analysis of TFE3 in XhCRC cells treated with DMSO (control) or CPT-11 (1 μM) for 24 h alone, or with Bis I (20 nM) for 1 h or with SB415286 (1 μM) for 12 h. **F** IP assays were performed to enrich HA-GSK3α, HA-GSK3β, and Flag-TFE3 in HEK293T cells. **G** QIP assays were performed to detect the phosphorylation and content of TFE3 with GSK3α or GSK3β overexpression in HEK293T cells. **H** QIP assays to detect the phosphorylation, content, and binding states to the 14-3-3 protein of wild-type (WT) TFE3 and mutant (S321A) TFE3 in HEK293T cells. **I** WT TFE3 and S321A TFE3 XhCRC cells were stained with VSVG-GFP, 8G5F11, and DAPI (nucleus) at 45 min. Scale bar: 20 μm.

Studies in lysosomes have revealed that active protein kinase C α (PKCα) phosphorylates and thereby inactivates glycogen synthase kinase 3β (GSK3β), resulting in reduced phosphorylation and degradation of transcription factor EB (TFEB) [51, 52]. An important question is whether this regulatory mechanism of TFEB is shared by other transcription factors that belong to the TFE family, including TFE3 [53]. Furthermore, the Golgi stress response leads to an increase in cytosolic calcium (Ca^2+^) [36], a typical condition for PKCα activation [54]. As measured by the Fluo-4 AM indicator [55], a significant increase in intracellular Ca^2+^ was observed in shNC cells treated with CPT-11, whereas GOLPH3 knockdown inhibited the increase in Ca^2+^ (Fig. 6A–C). CPT-11-induced Golgi dispersal increased the levels of phosphorylated PKCα and phosphorylated GSK3α/β, which were inhibited by GOLPH3 knockdown (Fig. 6D). The PKCα inhibitor Bisindolylmaleimide I (Bis I) [56] also decreased the degree of PKCα and GSK3α/β phosphorylation (Fig. 6D). Subsequently, inhibition of PKCα by Bis I or GSK3α/β by SB415286 revealed that the level of TFE3 was decreased by PKCα inhibition and increased by GSK3α/β inhibition (Fig. 6E). To further investigate the mechanisms through which GSK3α/β regulates TFE3, HA-GSK3α/β and Flag-TFE3 plasmids were constructed and transfected into HEK293T cells. Co-immunoprecipitation (Co-IP) verified the exogenous interaction between GSK3α/β and TFE3 (Fig. 6F). Quantitative IP (qIP) revealed that overexpression of GSK3α, but not GSK3β, resulted in the hyperphosphorylation of TFE3, which was associated with a decreased TFE3 level (Fig. 6G). TFE3 is phosphorylated at Ser321 and thus sequestered in the cytosol for degradation by 14-3-3 protein [57]. To further identify the phosphorylation site of TFE3 by GSK3α, a serine-to-alanine mutant Flag-TFE3 plasmid at Ser321 (S321A) was constructed. QIP indicated that the S321A mutation resulted in attenuation of TFE3 phosphorylation, impaired the association with 14-3-3 protein, and thus increased TFE3 level (Fig. 6H). Compared with the wild-type TFE3 group, the S321A mutation of TFE3 enhanced VSVG-GFP transport in XhCRC cells (Fig. 6I). In conclusion, these results demonstrate that GSK3α directly interacts with and phosphorylates TFE3 at Ser321. Golgi dispersal activates PKCα via elevated Ca^2+^, thereby inactivating GSK3α, resulting in reduced phosphorylation and an increased level of TFE3.

### Golgi dispersal increases the level of ABCG2 on the plasma membrane and reduces the intracellular drug concentration

**Fig. 7:**
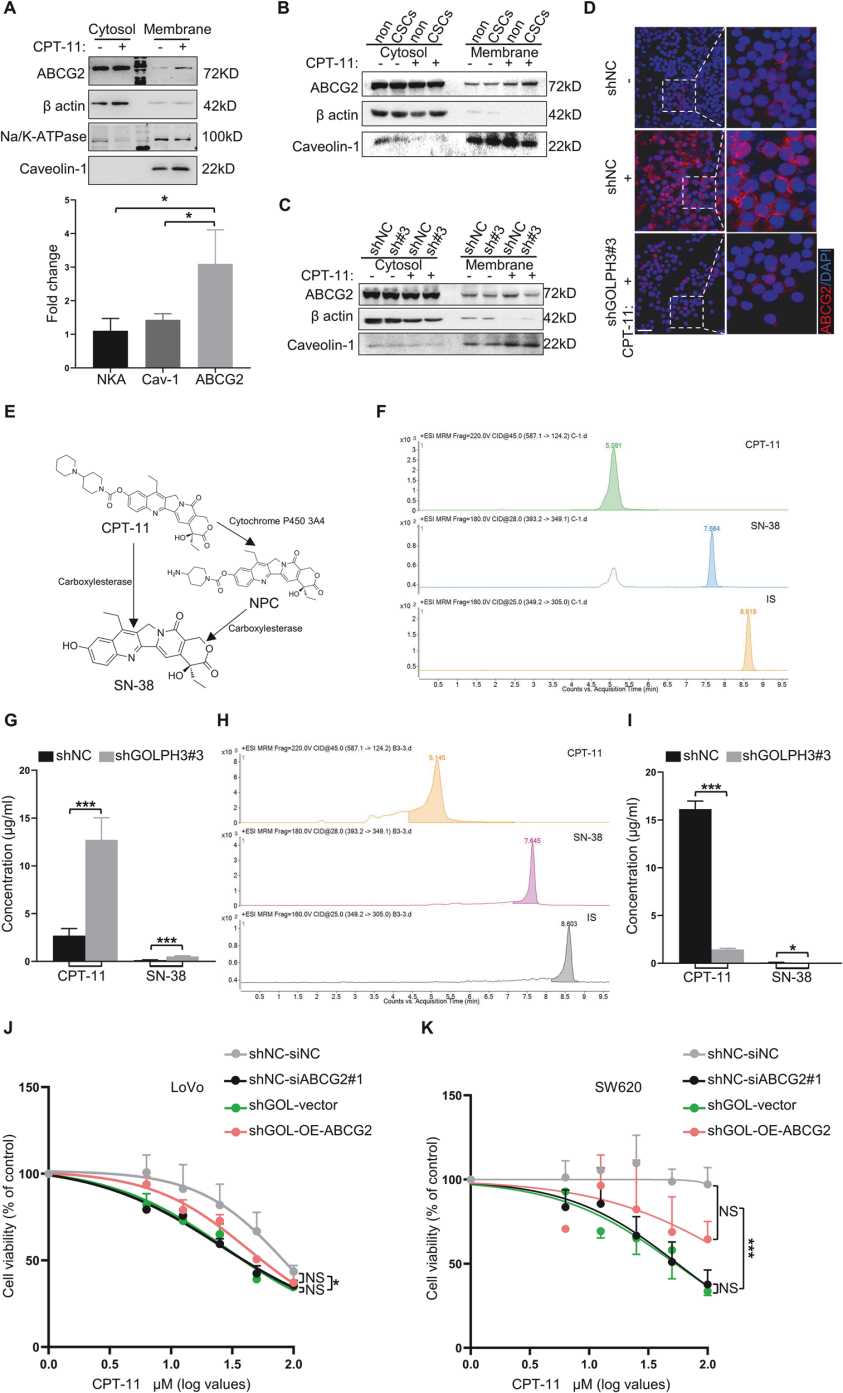
Golgi dispersal increases the level of ABCG2 on the plasma membrane and reduces the intracellular drug concentration. **A** Western blot analysis of the indicated molecules in XhCRC cells treated with DMSO (control) or CPT-11 (1 μM) for 24 h. The indicated molecules on the PM in the CPT-11 group were quantified relative to the control group. The data are shown as the mean ± SD (*n* = 3). ^*^P < 0.05. **B** Monolayer-cultured XhCRC non-CSCs (non) and sphere-derived XhCRC CSCs (CSCs) were treated with DMSO (control) or CPT-11 (1 μM) for 24 h. Western blot to detect ABCG2 in the cytosol and on the PM. **C** ShNC and shGOLPH3 XhCRC CSCs were treated with DMSO (control) or CPT-11 (1 μM) for 24 h. Western blot to detect ABCG2 in the cytosol and on the PM. **D** ShNC and shGOLPH3 XhCRC CSCs treated with DMSO (control) or CPT-11 (1 μM) for 24 h were stained with ABCG2 and DAPI (nucleus). Scale bar: 20 μm. **E** Schematic of the metabolic pattern of CPT-11 and SN38. **F**, **G** ShNC and shGOLPH3 XhCRC CSCs were treated with CPT-11 (20 μM) and SN-38 (20 μM) for 24 h. LC-MS analysis (**F**) and quantification (**G**) of intracellular CPT-11 and SN38 concentrations. The data are shown as the mean ± SD (*n* = 9). ^***^P < 0.001. **H,** **I** ShNC and shGOLPH3 XhCRC CSCs were treated as in **F**. LC-MS analysis (**H**) and quantification (**I**) of CPT-11 and SN38 concentrations in conditional medium. The data are shown as the mean ± SD (*n* = 3). ^*^P < 0.05, ^***^P < 0.001. Cell viability was measured relative to control in shNC-siNC, shNC-siABCG2, shGOL-vector, and shGOL-OE-ABCG2 LoVo (**J**) and SW620 (**K**) cells treated with indicated doses of CPT-11 for 24 h. The data are shown as the mean ± SD (*n* = 3). ^*^P < 0.05, ^***^P < 0.001. NS no significance.

Since Golgi dispersal enhances trafficking from Golgi to PM, we searched for drug resistance-associated proteins that are transported by Golgi apparatus. ABC family proteins, also known as ATP-binding cassette transporters, have an important impact on the drug resistance of cancer cells [58]. Furthermore, Golgi apparatus is responsible for the processing and transport of ABC family proteins [59]. Notably, CPT-11 and its active metabolite, SN-38, are particular substrates of ABCG2 [60, 61]. Thus, we investigated whether Golgi dispersal regulates ABCG2-dependent drug resistance. Compared with PM proteins (Na/K-ATPase and Caveolin-1), CPT-11-treated XhCRC cells significantly enhanced ABCG2 delivery to the PM (Fig. 7A), without increased transcription of *ABCG2* (Supplementary Fig. S6A). After treatment with CPT-11, sphere-derived XhCRC CSCs exhibited a significantly higher level of ABCG2 on the PM compared with non-CSCs (Fig. 7B). Knockdown of GOLPH3, which results in decreased Golgi dispersal, markedly reduced the level of ABCG2 on the PM (Fig. 7C, D). CPT-11 and SN-38 were subsequently detected and measured by liquid chromatography-mass spectrometry (LC-MS) (Fig. 7E). Interestingly, the intracellular concentrations of CPT-11 and SN-38 in shGOLPH3 cells were greater than those in control cells (Fig. 7F, G), whereas the concentrations of CPT-11 and SN-38 in the conditioned medium (CM) derived from the shGOLPH3 group were lower than those in the control group (Fig. 7H, I). To further clarify the role of ABCG2 in Golgi dispersal-mediated chemoresistance, we overexpressed ABCG2 in shGOLPH3 cells, in which Golgi dispersal was inhibited, and knocked down ABCG2 in shNC cells (Supplementary Fig. S6B, C). We assessed the viability of shNC-siNC, shNC-siABCG2, shGOL-vector, and shGOL-OE-ABCG2 LoVo and SW620 cells. It was found that knockdown of ABCG2 reduced the viability of shNC cells. Meanwhile, overexpression of ABCG2 in shGOLPH3 cells was able to rescue the reduced cell viability caused by Golgi dispersal inhibition (Fig. 7J, K). Notably, there was no significant difference between shNC-siNC and shGOL-OE-ABCG2 cells, or between shNC-siABCG2 and shGOL-vector cells (Fig. 7J, K). These results indicate that Golgi dispersal increases the transport of ABCG2 to the PM, which is responsible for the cytoprotective effect of Golgi dispersal.

## Discussion

In this study, we revealed that Golgi dispersal was triggered by DNA-damaging agents such as CPT-11 and DOXO. In addition, colorectal CSCs displayed greater Golgi dispersal than non-CSCs, based on GOLPH3. Moreover, Golgi dispersal was found to be responsible for cell survival following CPT-11 treatment. Furthermore, Golgi dispersal induced Golgi stress response via the PKCα/GSK3α/TFE3 pathway, which increased protein and vesicle transport, specifically ABCG2, and subsequently increased drug efflux.

Chemotherapy failure in CRC is caused by chemoresistance [4]. Understanding the cellular response to DNA damage is crucial for identifying the mechanisms of chemoresistance [62]. The response to DNA damage is largely determined by nuclear processes such as DNA repair and cell cycle [63]. However, the cytoplasmic response to DNA damage is poorly understood. Field and his colleagues discovered that Golgi dispersal was triggered by DNA damage [6, 9]. However, whether Golgi dispersal occurs in CRCs is uncertain. Consistent with previous studies, we demonstrated that Golgi dispersal was a specific cytoplasmic response to DNA-damaging agents in CRC cells, which was independent of cell apoptosis and promoted cell survival. Notably, Golgi dispersal was more active in colorectal CSCs than non-CSCs, highlighting the role of CSCs in chemoresistance.

The Golgi stress response is triggered by a blockage or overload of the secretory pathway, which activates the transcription of Golgi-related genes through the TFE3 pathway [19]. Golgi dispersal induced by the GOLPH3/MYO18A/F-actin pathway resembles the changes in Golgi morphology during the Golgi stress response [64]. Furthermore, GOLPH3 activation by phosphorylation increases the pulling force of Golgi and vesicle transport, which imitates the stimulus: an overload of the secretory pathway [6, 65]. GOLPH3 also acts as a potential receptor for the Golgi stress response [38]. Therefore, it was reasonable that Golgi dispersal boosted Golgi function through the Golgi stress response. We demonstrated that CPT-11-induced Golgi dispersal was a form of Golgi stress response, which increased the level of TFE3 and its downstream genes, including *STX3*, *WIPI1*, and *RAB20*. Interestingly, Golgi dispersal did not impede protein glycosylation, but rather significantly enhanced vesicle and protein transport.

TFE3 is essential for the Golgi stress response [41, 66]. However, the intricacies of the upstream pathway of TFE3 have yet to be fully investigated. Lysosomal-based studies have indicated a PKC-dependent mechanism regulating TFEB via the PKCα/GSK3β pathway [51, 52]. In the present study, we revealed that Golgi dispersal increased intracellular Ca^2+^, which activated PKCα. Activated PKCα phosphorylated and thus inactivated GSK3α. GSK3α interacted with and phosphorylated TFE3 at Ser321, which is sequestered in the cytosol by 14-3-3 proteins for degradation [57]. Inactivated GSK3α reduced the phosphorylation of TFE3, resulting in an increase in TFE3 levels.

ABCG2, a key member of the ABC transporter superfamily, plays an essential role in drug resistance [67]. Notably, ABCG2 requires the Golgi apparatus for processing and transporting to perform its activity [68, 69]. We revealed that ABCG2 is responsible for the cytoprotective effect of Golgi dispersal. Golgi dispersal increased the level of ABCG2 on the PM, which facilitated the efflux of CPT-11 and SN-38. Consistent with ABCG2, various proteins may be regulated by Golgi dispersal, ultimately contributing to the maintenance of cellular activity.

In conclusion, this study revealed the role of Golgi dispersal in regulating the chemoresistance of CRC cells. Mechanistically, Golgi dispersal was a special form of the Golgi stress response that enhanced the transport of proteins and vesicles. Strategies to relieve Golgi activity and inhibit Golgi stress responses could be used as therapeutic targets in CRC.

## Materials and methods

### Cell culture

The XhCRC cell line was obtained following previous procedures [32, 70]. The xenograft tumors were minced and subjected to enzymatic digestion at 37 °C for 30 min. A mixture of DMEM/F12 (Thermo Fisher Scientific; Waltham, MA, USA), collagenase IV (1.5 mg/mL) (Thermo Fisher Scientific), hyaluronidase (20 μg/mL) (Sigma-Aldrich; Louis, USA), penicillin (100 U/mL) (Thermo Fisher Scientific), and streptomycin (100 U/mL) (Thermo Fisher Scientific) was used for digestion. Isolated single cells were labeled with an EpCAM antibody conjugated with BUV737 (BD Biosciences; San Jose, CA, USA) and then purified by FACS (FACSAria II, BD Biosciences). The SW620, LoVo, XhCRC, and HEK293T cell lines were cultured in DMEM (Thermo Fisher Scientific) supplemented with 10% FBS (Thermo Fisher Scientific). Cells were treated with HBSS (Thermo Fisher Scientific) and HEPES (10 mM) (Thermo Fisher Scientific) for 4 h for starvation. Mycoplasma was routinely tested using a mycoplasma detection kit (Thermo Fisher Scientific). The clinical history of the human subject and the details of the cell lines are listed in Supplementary Table S1.

### Plasmid construction and transfection

The TOP-GFP plasmid was a gift from Ramesh Shivdasani (Addgene; Watertown, MA, USA) [30]. The TOP-GFP plasmid indicates tumor cells with high intrinsic Wnt activity, which is a distinguishing feature of colorectal CSCs [31]. The ts045-VSVG-EGFP plasmid was a gift from Jennifer Lippincott-Schwartz (Addgene) [48]. Cells infected with the ts045-VSVG-GFP plasmid were incubated in a cell culture incubator with 5% CO_2_ at 40 °C for 36 h for expression. Ts045-VSVG-GFP was retained in the ER at 40 °C. Transfected cells were then shifted to 32 °C to release VSVG-GFP from the ER to the Golgi and subsequently to the PM. GOLPH3-shRNAs, MYO18A-shRNAs, TFE3-shRNAs, Rab20-GFP, HA-tagged GSK3α/β, Flag-tagged TFE3, OE-ABCG2, and ABCG2-siRNAs were purchased from MiaoLingBio, China. The S321A mutant TFE3 was constructed using the Mut Express II Fast Mutagenesis Kit (Vazyme; China) according to the manufacturer’s instructions. CRC cells or HEK293T cells (1 ×10^6^ cells) were transfected with 4 μg of plasmid using 8 μL of ExFect Transfection Reagent (Vazyme) according to the manufacturer’s instructions. The details of the plasmids are listed in Supplementary Table S1.

### Lentivirus construction and transduction

400 μL of Opti-MEM (Thermo Fisher Scientific), 7.5 μg of psPAX2 plasmid (Addgene), 2.5 μg of pMD2.G plasmid (Addgene), 10 μg of TOP-GFP or shRNA plasmids (Addgene or MiaoLingBio), and 20 μL of ExFect Transfection Reagent (Vazyme) were combined and added to HEK293T cells. Media were collected from the HEK293T cells 48 h post-transfection and centrifuged at 2000 × *g*. The supernatant was subsequently used for lentiviral transduction. The cells were infected overnight with lentivirus and cultured with 2 μg/mL puromycin (Thermo Fisher Scientific) at 48 h post-transduction for 5 days. Infections were confirmed via western blot.

### Immunofluorescence (IF)

Cells were cultured in glass-bottomed Petri dishes overnight, fixed with 4% paraformaldehyde (PFA) for 10 min at room temperature, and permeabilized with 0.1% Triton X-100 (Sigma-Aldrich) for 5 min at room temperature. The cells were blocked with 5% bovine serum albumin (BSA) (Beyotime; China) in PBS. Primary antibodies were diluted with 5% BSA. After being incubated with primary antibodies overnight at 4 °C, cells were incubated with fluorophore-conjugated secondary antibodies for 2 h at room temperature. Samples were stained with DAPI (Sigma-Aldrich) for 10 min at room temperature, mounted with anti-fade mountant (Thermo Fisher Scientific), and visualized by a fluorescence microscope (Olympus BX53 or CKX41 microscope; OLYMPUS) or a confocal microscope (Olympus FV1000; OLYMPUS). The details of the antibodies are listed in Supplementary Table S1.

### Transmission electron microscopy (TEM)

Cells on plates were fixed with precooled 2% glutaraldehyde (pH 4.0–5.0) at 4 °C for 15 min and then scraped and centrifuged at 2000 × *g*. After rinsing with Milloning’s phosphate buffer (Sigma-Aldrich), the cell pellet was fixed at 4 °C with 1% osmic acid (pH 7.3–7.4) for 2 h and subsequently dehydrated with ethanol for 15 min. Samples were deposited onto Formvar-carbon-coated copper grids. The grids were stained with 2% uranyl acetate for 10 min and air-dried. The samples were observed with an FEI Tecnai T20 TEM (Philips Medical Systems; Amsterdam, Netherlands) at an accelerating voltage of 160 kV.

### Western blot and phos-tag SDS-PAGE

Cell pellets were lysed using NP40 lysis buffer (Thermo Fisher Scientific). Protein lysates were quantified using BCA assay kits (Thermo Fisher Scientific). For phos-tag SDS-PAGE to identify phosphorylated proteins, 50 μM Phos-tag acrylamide (APExBIO; Houston, TX, USA) and 100 μM MnCl_2_ were added to the 10% SDS-PAGE gels according to the manufacturer’s protocol. A total of 20 μg of protein sample was loaded onto 10% SDS-PAGE gels and transferred to 0.22-μm PVDF membranes (Sigma-Aldrich) using a wet transfer device. The membranes were blocked with 5% BSA in Tris-buffered saline solution supplemented with 0.05% Tween 20 (TBST) for 1 h and incubated with primary antibodies at 4 °C overnight, followed by incubation with horseradish peroxidase (HRP)-conjugated secondary antibodies (Abbkine; China). Finally, the membranes were imaged with an ECL substrate (Thermo Fisher Scientific) using an AlphaImager HP (Alpha Innotech; San Jose, CA, USA). The details of the antibodies are listed in Supplementary Table S1.

### Flow cytometric analysis and fluorescence-activated cell sorting (FACS)

For flow cytometric analysis, cells were collected and stained with Annexin V-PE/7-AAD apoptosis detection kits (Vazyme) or Annexin V-FITC/PI apoptosis detection kits (Vazyme) according to the manufacturer’s protocol. Cells were analyzed with a FACSAria II Cell Sorter (BD Biosciences). The flow cytometer was set at 560 and 488 nm (excitation wavelengths) to detect fluorescence. Cells stained with Fluo-4 AM or WGA were collected and analyzed by the FACSAria II Cell Sorter. The flow cytometer was set at 488 nm (excitation wavelengths) to detect fluorescence. Analyses were performed using FACSDiva software (BD Biosciences). For FACS, cells transfected with TOP-GFP lentivirus were collected and analyzed by the FACSAria II Cell Sorter. The flow cytometer was set at 488 nm (excitation wavelengths) to detect fluorescence. Analyses were performed using FACSDiva software, and the top 5% (TOP-GFP^high^) or bottom 5% (TOP-GFP^low^) of cells were sorted based on the GFP signal.

### Cell viability analysis

Cell viability was assessed using a cell counting kit-8 (CCK-8; MedChemExpress; Monmouth Junction, NJ, USA). Cells in 96-well plates were treated with different doses of CPT-11 (MedChemExpress). After 24 h, 10 μL of CCK-8 solution was added to each well, and the plate was incubated at 37 °C for 2 h. Finally, the cell viability was assessed by scanning with a microplate reader at 450 nm.

### In vivo assays

Four-week-old female NOD/Scid mice (GemPharmatech; China) were randomly divided into groups (3–5 mice per group). Mice were maintained in a temperature-controlled system at 22 °C with a 12-h dark/light cycle. Cells (1 ×10^6^ cells) in 100 μL of PBS mixed with Matrigel (BD Biosciences) at a 1:1 ratio were subcutaneously implanted. After the tumor had reached 30–60 mm^3^, intraperitoneal injection of CPT-11 (100 mg/kg; MedChemExpress) was performed every 7 days. 6–7 weeks later, all the mice were euthanized by CO_2_ inhalation (30% vol/min). Tumor volumes were imaged and calculated using the formula length × width^2^/2. The measurements were performed blindly.

### Immunohistofluorescence (IHF)

Tumors embedded in paraffin blocks were deparaffinized and hydrated using ethanol. After microwave antigen retrieval in Dako target retrieval solution (Agilent; Santa Clara, CA, USA), the slides were incubated in a 0.3% hydrogen peroxide solution for 15 min at room temperature. Next, samples were blocked with serum-free protein blocking solution (Agilent) and incubated with the corresponding primary antibodies at 4 °C overnight. Slides were then incubated with fluorophore-conjugated secondary antibodies for 2 h and DAPI for 10 min at room temperature. Samples were visualized by a fluorescence microscope (Olympus BX53 or CKX41 microscope; OLYMPUS) or a confocal microscope (Olympus FV1000; OLYMPUS). The details of the antibodies are listed in Supplementary Table S1.

### Isolation of nucleus- or membrane-associated proteins

Nucleus-associated proteins were extracted using a NE-PER extraction kit (Thermo Fisher Scientific). 200 μL of ice-cold cytoplasmic extraction reagent I was added to the cell pellet and incubated on ice for 10 min. After adding 11 μL of ice-cold cytoplasmic extraction reagent II and incubating on ice for 1 min, the tube was centrifuged at 4 °C and 16,000 × *g* for 10 min, after which cytoplasmic proteins were enriched in the supernatant. The insoluble fraction, which contained nuclei, was suspended in 100 μL of ice-cold nuclear extraction reagent and incubated on ice for 40 min. The tube was subsequently centrifuged at 4 °C and 16,000 × *g* for 10 min, after which nucleus-associated proteins were enriched in the supernatant. Membrane-associated proteins were extracted using a Mem-PER extraction kit (Thermo Fisher Scientific). 750 μL of permeabilization buffer was added to the cell pellet, which was incubated on ice for 10 min. The tube was centrifuged at 4 °C and 16,000 × *g* for 15 min, after which cytoplasmic proteins were enriched in the supernatant. The insoluble fraction was suspended in 500 μL of solubilization buffer and incubated on ice for 30 min. The tube was then centrifuged at 4 °C and 16,000 × *g* for 15 min, after which membrane-associated proteins were enriched in the supernatant.

### Reverse transcription quantitative (RT-q) PCR analysis

Total RNA was extracted from CRC cells using TRIzol (TaKaRa; Shiga, Japan), and cDNA was synthesized using PrimeScript RT Master Mix (Takara) according to the manufacturer’s protocol. qPCR was performed using SYBR Green PCR Master Mix (Takara) on an ABI PRISM 7300 Sequence Detection System (Applied Biosystems; Foster City, CA, USA). The expression data were uniformly standardized to the internal control gene *GAPDH*, and the relative expression levels were assessed using the ΔΔCt method. The details of the primers are listed in Supplementary Table S2.

### Co-immunoprecipitation (Co-IP) and quantitative immunoprecipitation (qIP)

Cells were lysed in an NP40 solution on ice for 30 min. After centrifugation at 4 °C and 16,000 × *g* for 10 min, the supernatant protein concentration was determined via a BCA assay kit (Thermo Fisher Scientific) and diluted to 1 μg/μL with NP40. 20 μL of anti-HA magnetic beads or anti-Flag magnetic beads (MedChemExpress) were added to 700 μL of protein supernatant. The mixture was incubated at 4 °C overnight. The beads were collected and then boiled in 70 μL of SDS loading buffer at 100 °C for 10 min. A total of 15 μL per sample was loaded onto 10% SDS-PAGE gels for western blot.

### Conditioned medium (CM) preparation

CM was generated from shNC or shGOLPH3 CRC cells. Cells were rinsed with PBS and incubated with fresh DMEM (Thermo Fisher Scientific) supplemented with CPT-11 (MedChemExpress) and SN-38 (MedChemExpress) at 37 °C for 24 h. CM was collected and filtered through a 0.22 µm filter (Millipore, Billerica, MA, USA) to eliminate cellular debris.

### Liquid chromatography-mass spectrometry (LC-MS)

The cell pellet and CM were disrupted using an ultrasonic cell disrupter system (Thermo Fisher Scientific). Aliquots of 200 μL of acetonitrile (0.5%) were used as the extraction agent. The samples were vortexed for 3 min and then centrifuged at 16,000 × *g* for 10 min. The supernatant was filtered through a 0.22 μm microporous membrane. Samples of cleared supernatant (2 μL) were injected via a ULTRA high-performance liquid chromatography system (UPLC) (Waters Corporation; Milford, MA, USA) and separated using an XDB-C18 column (3.5 μm particle size, 2.1 × 150 mm; Agilent) maintained at 55 °C. A SCIEX Triple Quad 3500 LC-MS/MS System (SCIEX; Framingham, MA, USA) with MultiQuant 3.0.2 software (SCIEX) was used for the MS analysis.

### Sphere formation assays

The sphere formation assay was conducted as previously described [33]. CRC cells were plated in 6-well ultralow attachment plates (Corning; NY, USA) and resuspended in serum-free DMEM/F12 (Thermo Fisher Scientific) supplemented with B27 (1×) (Thermo Fisher Scientific), EGF (20 ng/mL) (Sigma-Aldrich), and human basic-FGF (20 ng/mL) (Thermo Fisher Scientific). CRC cells were cultured in a 5% CO_2_ cell culture incubator at 37 °C for 5–7 days, and tumor spheres were counted under a phase-contrast microscope.

### Statistical analysis

Statistical significance was determined with GraphPad Prism 8.0 (GraphPad Software; San Diego, CA, USA). Data are presented as the mean ± SD, unless otherwise stated. The data were analyzed using Student’s *t* test for two groups and ANOVA followed by Tukey’s test for multiple groups. *P* < 0.05 was considered to be statistically significant.

### Supplementary information

Supplementary Materials

Original Western Blots

## Data Availability

The data that supports the findings of this study is available from the corresponding author on reasonable request.
