# Supplementary material for: Golgi dispersal in cancer stem cells promotes chemoresistance of colorectal cancer via the Golgi stress response
Source: Cell Death Dis. 2024 Jun 15;15(6):417. doi: 10.1038/s41419-024-06817-0 (PMC11180190; doi:10.1038/s41419-024-06817-0)
Supplement: Supplementary file 1 — Supplementary Materials [file 41419_2024_6817_MOESM1_ESM.docx]

**Supplementary figures and tables: 6 figures and 2 tables.**

**
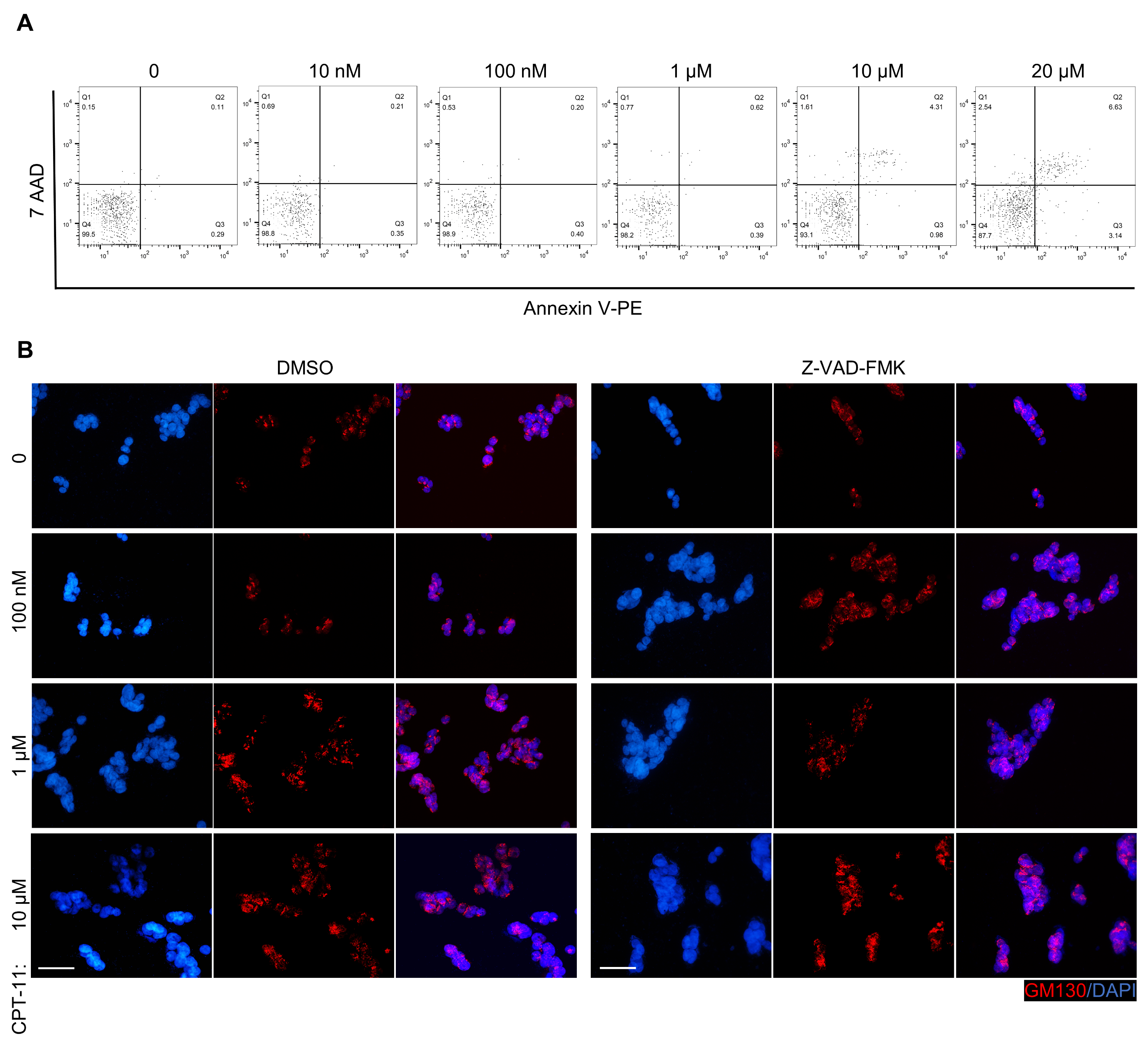
**

**Fig. S1 Golgi dispersal is independent of apoptosis.** **A** XhCRC cells were treated with the indicated doses of CPT-11 for 24 h. The percentage of apoptotic cells (Q2 + Q3) was measured by flow cytometry. **B** XhCRC cells were treated with the indicated doses of CPT-11 for 24 h, but also pretreated with DMSO or Z-VAD-FMK (40 μM) for 30 min. Cells were stained with GM130 (cis-Golgi) and DAPI (nucleus). Scale bar: 20 μm.

**
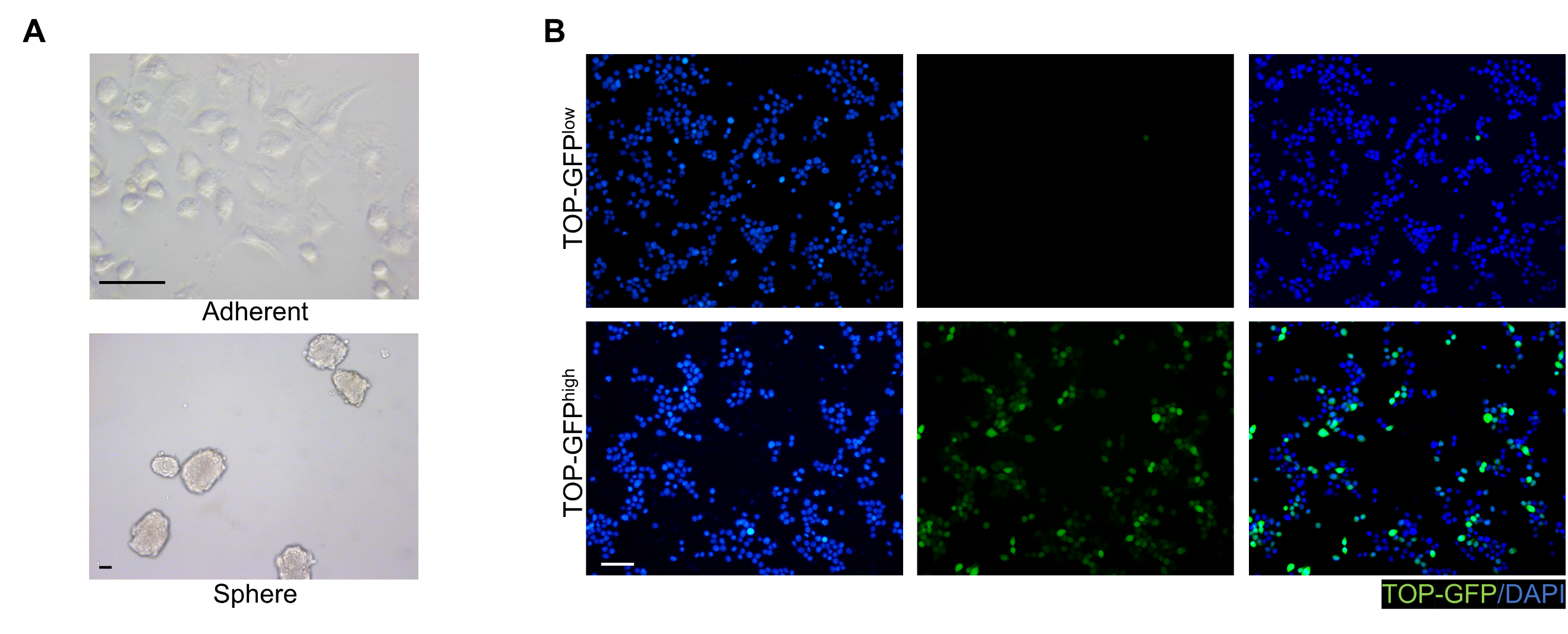
**

**Fig. S2 CSCs are enriched from CRC bulk cells.** **A** Non-CSCs were enriched in adherent (monolayer-cultured) XhCRC cells. Scale bar: 20 μm. CSCs were enriched in spheres (tumorsphere formation). Scale bar: 50 μm. **B** Sorted TOP-GFP^low^ and TOP-GFP^high^ XhCRC cells were stained with TOP-GFP and DAPI (nucleus). Scale bar: 20 μm.

**
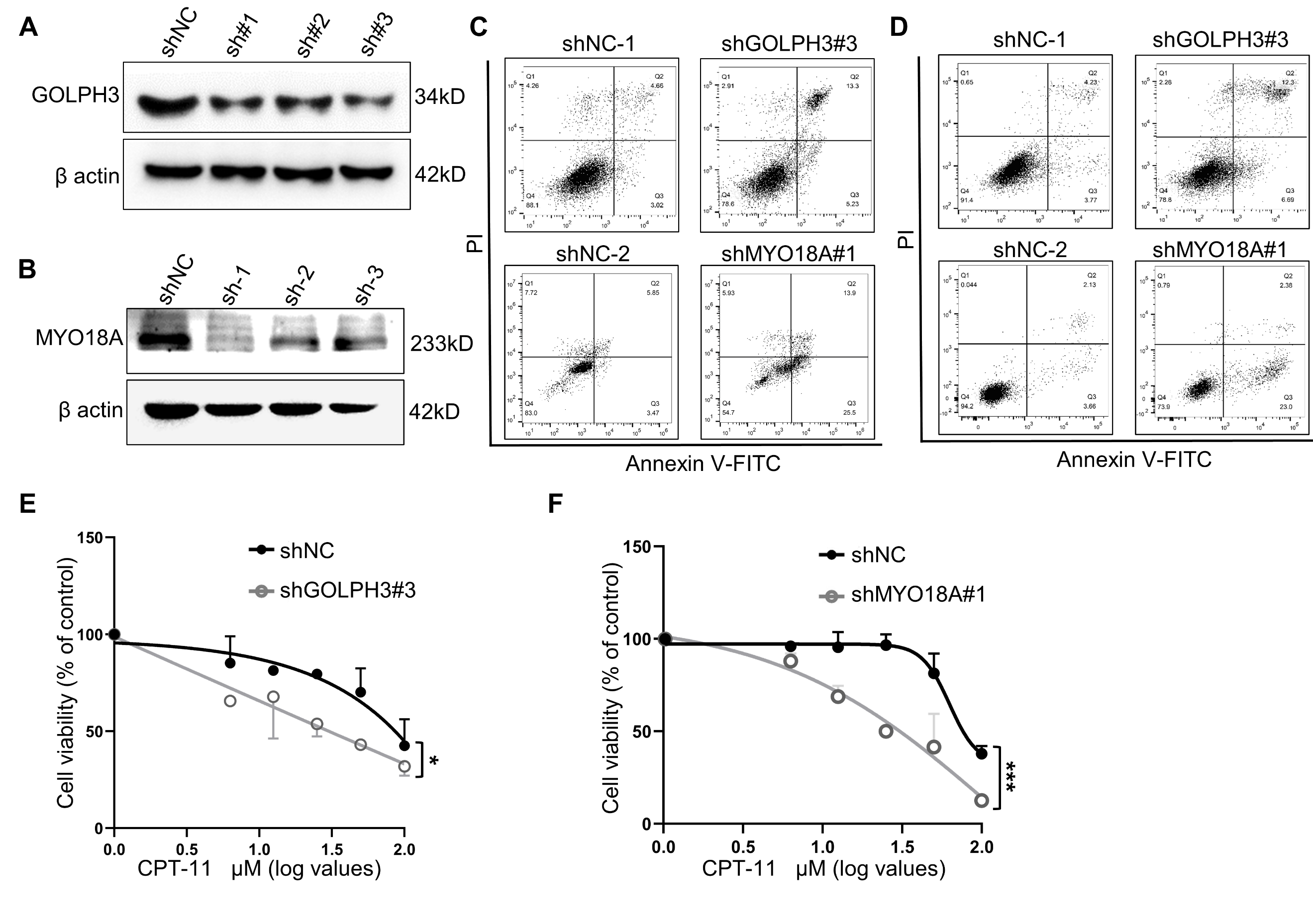
**

**Fig. S3 GOLPH3 and MYO18A are required for cell survival after CPT-11.** **A** Knockdown of GOLPH3 in XhCRC cells by GOLPH3-shRNAs. **B** Knockdown of MYO18A in XhCRC cells by MYO18A-shRNAs. **C** and **D** ShNC, shGOLPH3, and shMYO18A sphere-derived XhCRC (C) and SW620 (D) cells were treated with CPT-11 (20 μM) for 24 h. The percentage of apoptotic cells (Q2 + Q3) was measured by flow cytometry. **E** and **F** Cell viability was measured relative to control in shNC, shGOLPH3, and shMYO8A sphere-derived SW620 cells treated with indicated doses of CPT-11 for 24 h. The data are shown as the mean ± SD (*n* = 3). ^*^*P* < 0.05, ^***^*P* < 0.001.

**
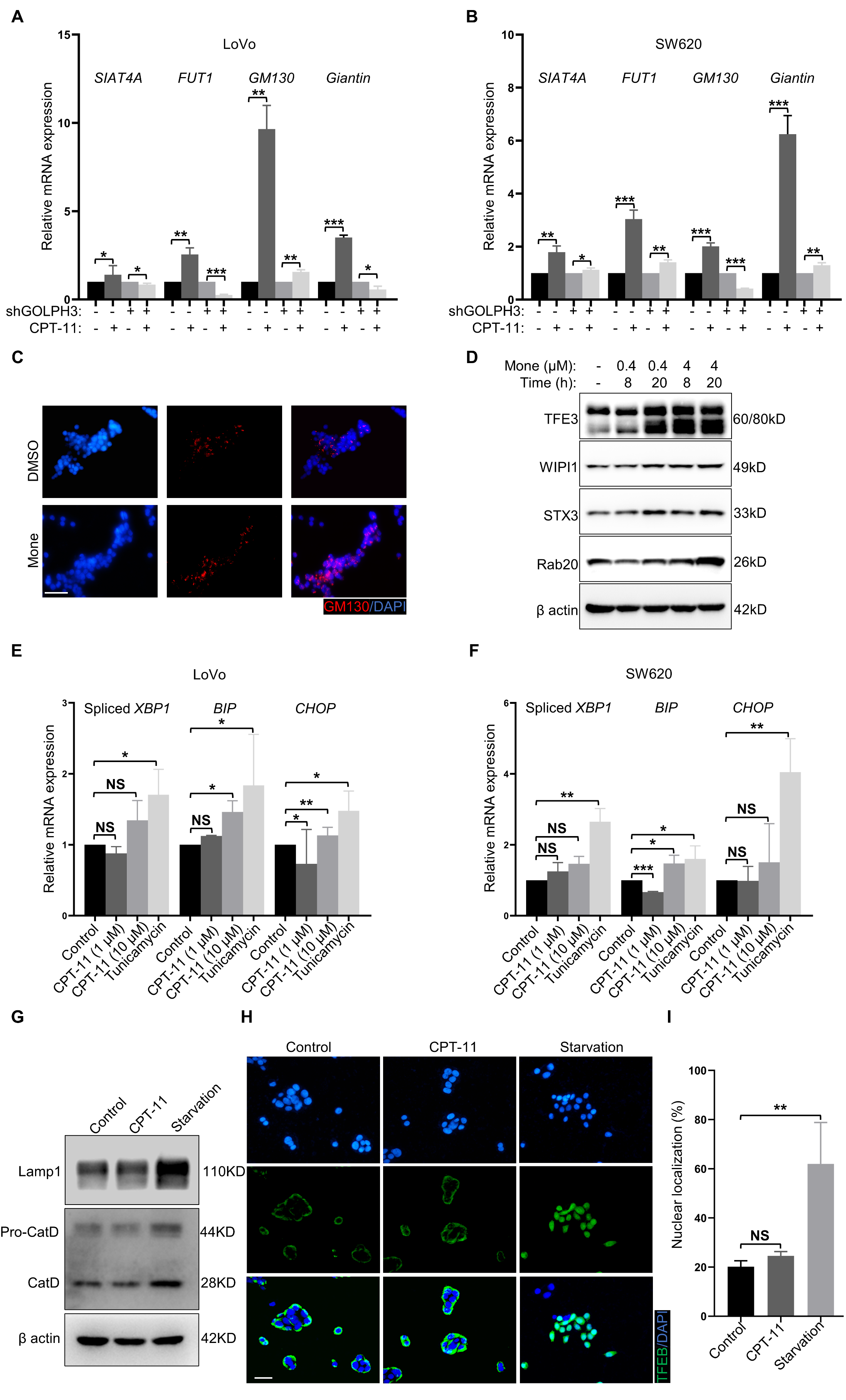
**

**Fig. S4 CPT-11-induced Golgi dispersal is a specific form of Golgi stress response.** **A** and **B** Expression levels of indicated mRNAs in shNC and shGOLPH3 LoVo (A) and SW620 (B) cells treated with DMSO (control) or CPT-11 (1 μM) for 24 h. The data are shown as the mean ± SD (*n* = 3). ^*^*P* < 0.05, ^**^*P* < 0.01, ^***^*P* < 0.001. **C** XhCRC cells treated with monensin (mone) (0.4 μM) for 20 h were stained with GM130 (cis-Golgi) and DAPI (nucleus). Scale bar: 20 μm. **D** Western blot analysis of the indicated molecules in XhCRC cells treated with DMSO (control) or indicated doses and durations of mone. **E** and **F** Expression levels of indicated mRNAs in LoVo (E) and SW620 (F) cells treated with DMSO (control), CPT-11 (1 μM), CPT-11 (10 μM), or tunicamycin (2 μM) for 24 h. The data are shown as the mean ± SD (*n* = 3). ^*^*P* < 0.05, ^**^*P* < 0.01, ^***^*P* < 0.001. NS, no significance. **G** Western blot analysis of indicated proteins in XhCRC cells treated with DMSO (control), CPT-11 (1 μM, 24 h), or starvation (HBSS, 4 h). **H** XhCRC cells treated with DMSO (control), CPT-11 (1 μM, 24 h), or starvation (HBSS, 4 h) were stained with TFEB and DAPI (nucleus). Scale bar: 20 μm. **I** Quantification of TFEB nuclear localization. The data are shown as the mean ± SD (*n* = 3). ^**^*P* < 0.01. NS, no significance.

**
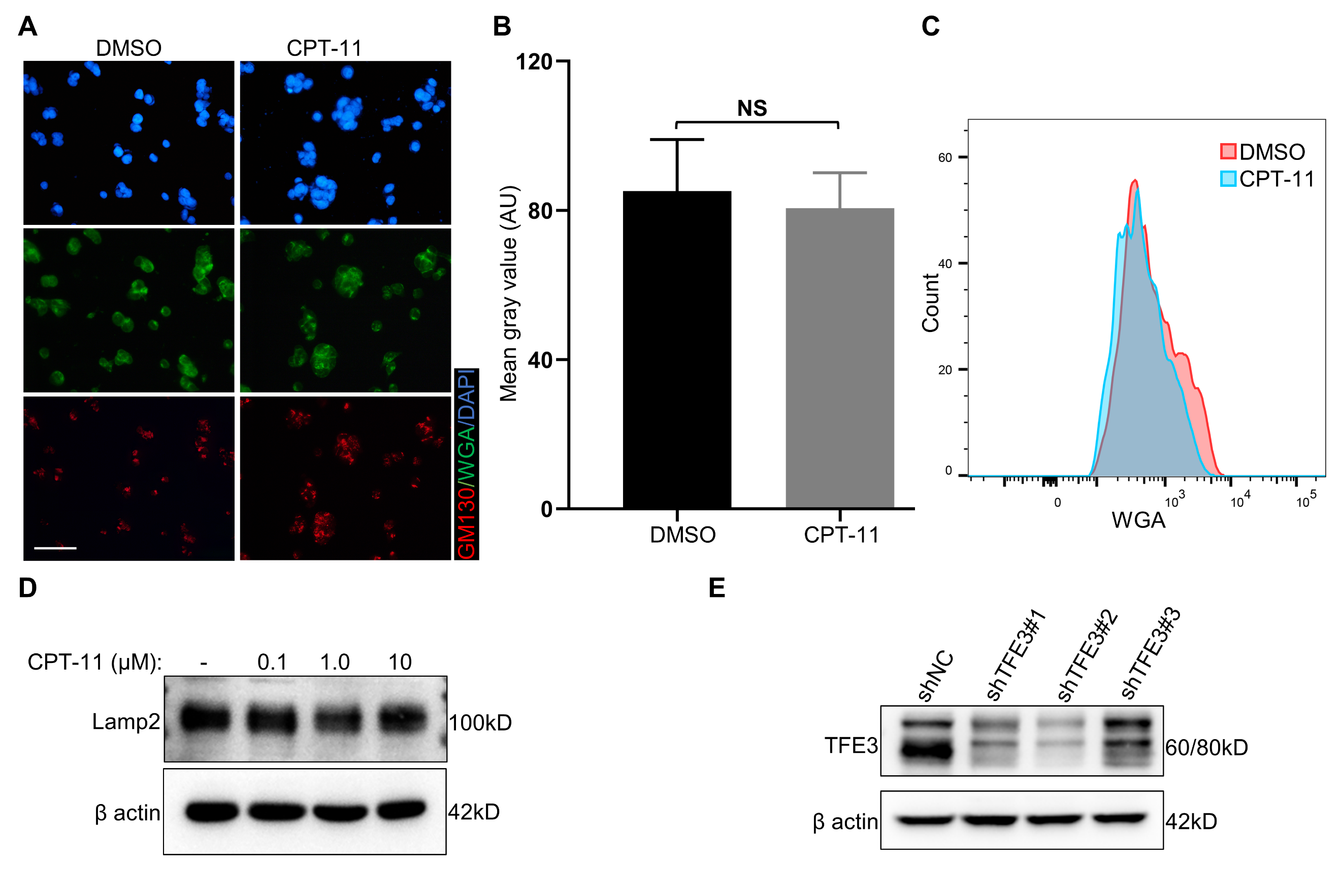
**

**Fig. S5 Golgi dispersal has no impact on protein glycosylation.** **A** XhCRC cells treated with DMSO (control) or CPT-11 (1 μM) for 24 h were stained with GM130 (cis-Golgi), WGA, and DAPI (nucleus). Scale bar: 20 μm. **B** The mean gray value of WGA was measured. The data are shown as the mean ± SD (*n* = 3). NS, no significance. **C** The signal of WGA was measured by flow cytometry. **D** XhCRC cells were treated with the indicated doses of CPT-11 for 24 h. The mobility of Lamp2 on the gel was assessed by western blot. **E** Knockdown of TFE3 in XhCRC cells by TFE3-shRNAs.


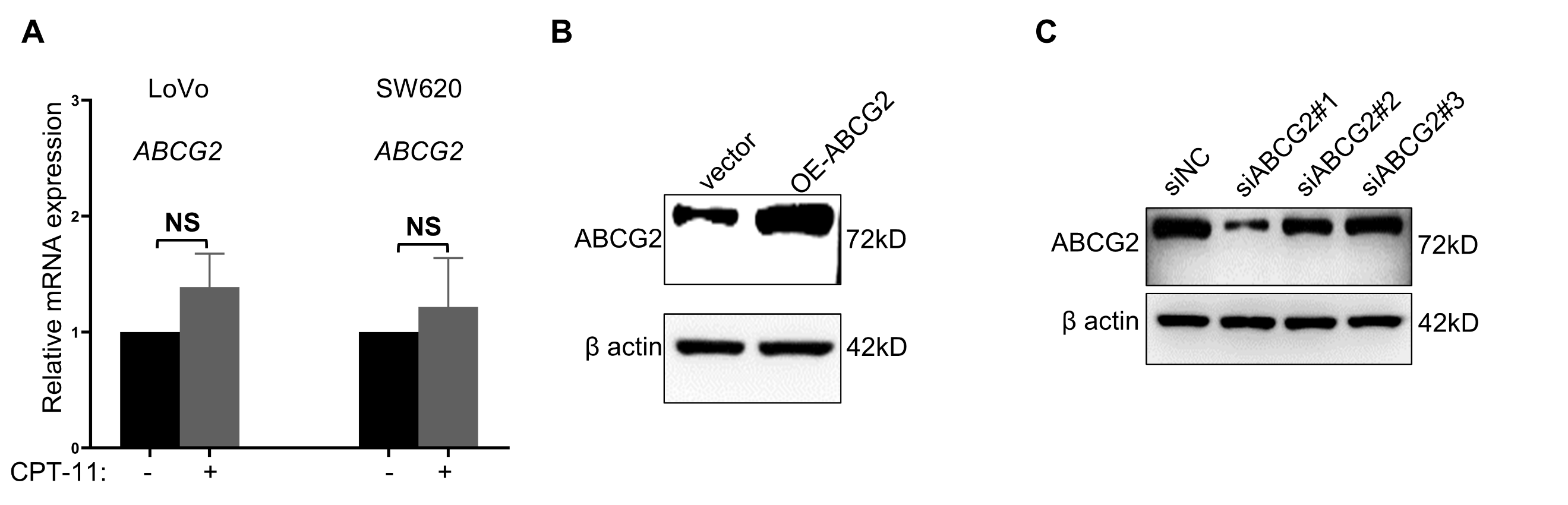


**Fig. S6 ABCG2 expression was verified by RT-qPCR and western blot. A** Expression of *ABCG2* in LoVo and SW620 cells treated with DMSO (control) or CPT-11 (1 μM) for 24 h. The data are shown as the mean ± SD (*n* = 3). NS, no significance. **B** Overexpression of ABCG2 in shGOLPH3 cells. **C** Knockdown of ABCG2 in shNC cells by ABCG2-siRNAs.

**Table** **S1. MATERIALS.**

| **Reagents or Resources** | **Source** | **Identifier** | **Dilution** |
| --- | --- | --- | --- |
| **Antibodies** | | | |
| BUV737 Anti-Human EPCAM  GM130  Phospho-Histone H2A.X (Ser139)  Phospho-DNA-PK (Ser2056)  VSV-G (8G5F11)  GOLPH3  MYO18A  ABCG2  ABCG2  TFE3  TFEB  Rab20  STX3  WIPI1  Lamp2  Lamp1  Cathepsin D  Sox2  CD133  Notch1  Caspase-3  Cleaved caspase-3  PKCα  Phospho-PKCα/β (Thr638/641)  GSK3α  Phospho-GSK3α (Ser21)  GSK3β  Phospho-GSK3β (Ser9)  Phospho-Ser/Thr  14-3-3  Caveolin-1  Na/K-ATPase  Histone H3  GAPDH  β actin  Goat Anti-Mouse IgG, DyLight 488  Goat Anti-Mouse IgG, Cy3  Goat Anti-Rabbit IgG, DyLight 488  Goat Anti-Rabbit IgG, Cy3  Goat Anti-Mouse IgG, HRP  Goat Anti-Rabbit IgG, HRP | BD Biosciences  Abcam  Cell Signaling Technology  Abcam  Kerafast  Proteintech  Affinity  ABclonal  Abcam  ATLAS  Abcam  Abclonal  ABclonal  ABclonal  Cell Signaling Technology  Abcam  Abcam  Cell Signaling Technology  Abcam  ABclonal  Cell Signaling Technology  Cell Signaling Technology  ABclonal  Cell Signaling Technology  Cell Signaling Technology  ABclonal  Cell Signaling Technology  ABclonal  ABclonal  ABclonal  Cell Signaling Technology  ABclonal  Beyotime  Abcam  Abcam  Abbkine  Abbkine  Abbkine  Abbkine  Abbkine  Abbkine | Cat#748382, RRID:AB_2872801  Cat#ab52649, RRID:AB_880266  Cat#80312, RRID:AB_2799949  Cat#ab124918, RRID:AB_11001004  Cat#EB0010, RRID:AB_2811223  Cat#19112-1-AP, RRID:AB_2113342  Cat#DF12298, RRID:AB_2845103  Cat#A5661, RRID:AB_2766421  Cat#ab3380, RRID:AB_303758  Cat#HPA023881, RRID:AB_1857931  Cat#ab270604  Cat#A17725, RRID:AB_2771902  Cat#A3712, RRID:AB_2863127  Cat#A9600, RRID:AB_2863733  Cat#49067, RRID:AB_2799349  Cat#ab24170, RRID:AB_775978  Cat#ab6313, RRID:AB_305416)  Cat#3579, RRID:AB_2195767  Cat#ab16518, RRID:AB_302419  Cat#A19090, RRID:AB_2862582  Cat#9662, RRID:AB_331439  Cat#9661, RRID:AB_2341188  Cat#A13342, RRID:AB_2760199  Cat#9375, RRID:AB_2284224  Cat#4337, RRID:AB_10859910  Cat#AP0582, RRID:AB_2771150  Cat#12456, RRID:AB_2636978  Cat#AP1088, RRID:AB_2863959  Cat#AP0893, RRID:AB_2770782  Cat#A9151, RRID:AB_2863673  Cat#3267, RRID:AB_2275453  Cat#A12405, RRID:AB_2759249  Cat#AH433, RRID:AB_2617171  Cat#ab9484, RRID:AB_307274  Cat#ab6276, RRID:AB_2223210  Cat#A23210, RRID:AB_2923050  Cat# A22210, RRID:AB_2923040  Cat# A23220, RRID:AB_2737289  Cat#A22220, RRID:AB_2923041  Cat#A21010, RRID:AB_2728771  Cat#A21020, RRID:AB_2876889 | 0.2 mg/mL  1:1 000/100  1:100  1:100  1:200  1:1 000  1:1 000  1:1 000  1:200  1:1 000  1:200  1:1 000  1:1 000  1:1 000  1:1 000  1:1 000  1:1 000  1:1 000  1:1 000  1:1 000  1:1 000  1:1 000  1:1 000  1:1 000  1:1 000  1:1 000  1:1 000  1:1 000  1:500  1:1 000  1:500  1:1 000  1:500  1:1 000  1:1 000  1:100  1:100  1:100  1:100  1:5 000  1:5 000 |
| **Bacterial and virus strains** | | | |
| TOP-GFP  ts045-VSVG-GFP  GOLPH3-shRNA  MYO18A-shRNA  TFE3-shRNA  Rab20-GFP  HA-GSK3α  HA-GSK3β  Flag-TFE3  OE-ABCG2  ABCG2-siRNA  psPAX2  pMD2.G | Addgene  Addgene  MiaoLingBio  MiaoLingBio  MiaoLingBio  MiaoLingBio  MiaoLingBio  MiaoLingBio  MiaoLingBio  MiaoLingBio  MiaoLingBio  Addgene  Addgene | Cat#35489, RRID:Addgene_35489  Cat#11912, RRID:Addgene_11912  N/A  N/A  N/A  N/A  Cat#P35439  N/A  N/A  N/A  N/A  Cat#12260, RRID:Addgene_12260  Cat#12259, RRID:Addgene_12259 |  |
| **Chemicals, peptides, and recombinant proteins** | | | |
| DMEM/F12  Opti-MEM  Collagenase IV  Hyaluronidase  Penicillin-Streptomycin  DMEM  HBSS  HEPES  FBS  BSA  B27  Human EGF  Human basic-FGF  DAPI  Phos-tag acrylamide  ExFect Transfection Reagent  Puromycin  Irinotecan  SN-38  Doxorubicin  5-Fluorouracil  NU7026  Z-VAD-FMK  Tunicamycin  Monesin  WGA  Brefeldin A (BFA)  Fluo-4 AM  Bisindolylmaleimide Ⅰ  SB415286  Anti-HA magnetic beads  Anti-Flag magnetic beads | Gibco  Gibco  Gibco  Sigma-Aldrich  Gibco  Gibco  Gibco  Gibco  Gibco  Beyotime  Gibco  Sigma-Aldrich  Gibco  Sigma-Aldrich  APExBIO  Vazyme  Thermo Fisher  MedChemExpress  MedChemExpress  MedChemExpress  MedChemExpress  MedChemExpress  MedChemExpress  MedChemExpress  MedChemExpress  Invitrogen  MedChemExpress  MedChemExpress  MedChemExpress  MedChemExpress  MedChemExpress  MedChemExpress | Cat#11320033  Cat#31985062  Cat#17104019  Cat#H3506  Cat#15140122  Cat#11965082  Cat#14025076  Cat#15630106  Cat#10099158  Cat#ST023  Cat#A1486701  Cat#SRP3027  Cat#PHG0264  Cat#d9542  Cat#F4002  Cat#T101-01  Cat#A1113803  Cat#HY-16562  Cat#HY-13704  Cat#HY-15142A  Cat#HY-90006  Cat#HY-15719  Cat#HY-16658B  Cat#HY-A0098  Cat#HY-N4302  Cat#W11261  Cat#HY-16592  Cat#HY-101896  Cat#HY-13867  Cat#HY-15438  Cat#HYK0201  Cat#HYK0207 | N/A  N/A  1.5 mg/mL  20 μg/mL  100 U/mL  N/A  N/A  10 mM  10%  50 g/L  1 ×  20 ng/mL  20 ng/mL  0.5 μg/mL  50 μM  N/A  2 μg/mL  N/A  20 μM  20 nM  1 μM  10μM  40 μM  2 μM  N/A  5 nM  2 μM  1 μM  20 nM  1 μM  N/A  N/A |
| **Critical commercial assays** | | | |
| Mycoplasma Detection Kit | Applied Biosystems | Cat#4460623 |  |
| BCA Assay Kit | Thermo Scientific | Cat#A55864 |  |
| Annexin V-PE/7-AAD Kit | Vazyme | Cat#A213-01 |  |
| Annexin V-FITC/PI Kit | Vazyme | Cat#A211-01 |  |
| Mut Express II Fast Mutagenesis Kit | Vazyme | Cat#C214-01 |  |
| Cell Counting Kit-8 | MedChemExpress | Cat#HY-K0301 |  |
| NE-PER Extraction Kit | Thermo Scientific | Cat#78833 |  |
| Mem-PER Extraction Kit | Thermo Scientific | Cat#89842 |  |
| **Experimental models: Cell lines** | | | |
| SW620 | ATCC | Cat#CCL-227, RRID:CVCL_0547 |  |
| LoVo | ATCC | Cat#CCL-229, RRID:CVCL_0399 |  |
| HEK293T | ATCC | Cat#CRL-3216, RRID:CVCL_0063 |  |
| **Experimental models: Organisms/strains** | | | |
| NOD/Scid mice | GemPharmatech | Cat#T001492 |  |
| DH5α Chemically Competent E.coli | TSINGKE | Cat#TSC-C14 |  |
| **Patient** | **Gender/Age** | **TNM** | **Stage** |
| XhCRC | F/47 | T4N2M0 | III |
| **Software and algorithms** | | | |
| FACSDiva software |  | BD Biosciences |  |
| FlowJo |  | BD Biosciences |  |
| MultiQuant |  | SCIEX |  |
| SnapGene |  | Dotmatics |  |
| GraphPad Prism |  | Dotmatics |  |

**Table** **S2. Primer sequences of RT-qPCR.**

| **Genes** | **Forward Oligo sequences (5’ to 3’)** | **Reverse Oligo sequences (5’ to 3’)** |
| --- | --- | --- |
| *STX3* | TCGGCAGACCTTCGGATTC | TCCTCATCGGTTGTCTTTTTGC |
| *WIPI1* | AACAGGTCTATGTGCTCTCTCT | CTCATGGGCAGCAATAGTGC |
| *RAB20* | CTATGATGTGAATCACCGGCA | GGTCCCCAGCGTCCATATTG |
| *GM130* | CCCGCGATGTCGGAAGAAA | GCATTGTCCTTGGGTGTATCCT |
| *Giantin* | GTGCTCAGGTCGTTGACTTG | TGTTTCCACAGTGTTCCTCAAA |
| *SIAT4A* | ACAGTCACGACTTTGTCCTCA | TGGTCTTGGTCCCAACATCAG |
| *FUT1* | TGGACTGTCTACCCCAATGG | CAGGGTGATGCGGAATACCG |
| Spliced *XBP1* | GCTGAGTCCGCAGCAGGT | CTGGGTCCAAGTTGTCCAGAAT |
| *BIP* | CACGGTCTTTGACGCCAAG | CCAAATAAGCCTCAGCGGTTT |
| *CHOP* | GGAAACAGAGTGGTCATTCCC | CTGCTTGAGCCGTTCATTCTC |
| *ABCG2* | ACGAACGGATTAACAGGGTCA | CTCCAGACACACCACGGAT |
| *GAPDH* | GGAGCGAGATCCCTCCAAAAT | GGCTGTTGTCATACTTCTCATGG |
