# Supplementary figures and images for: Golgi dispersal in cancer stem cells promotes chemoresistance of colorectal cancer via the Golgi stress response
Source: Cell Death Dis. 2024 Jun 15;15(6):417. doi: 10.1038/s41419-024-06817-0 (PMC11180190; doi:10.1038/s41419-024-06817-0)

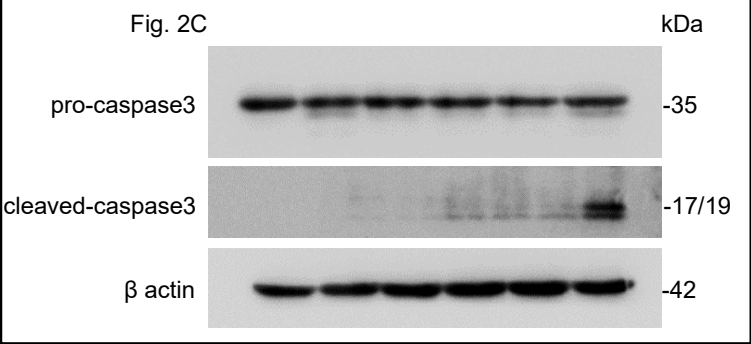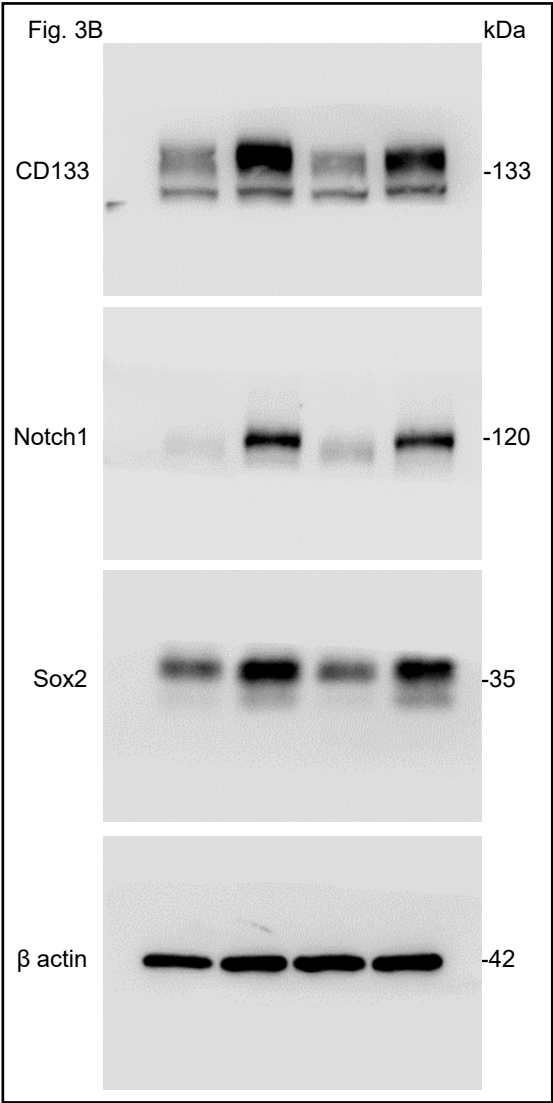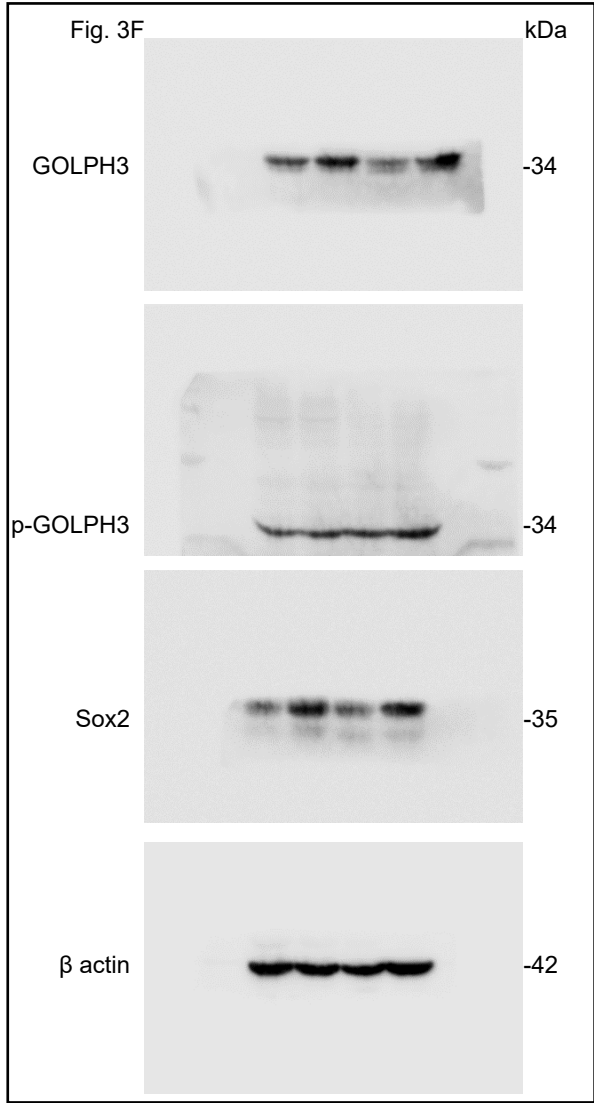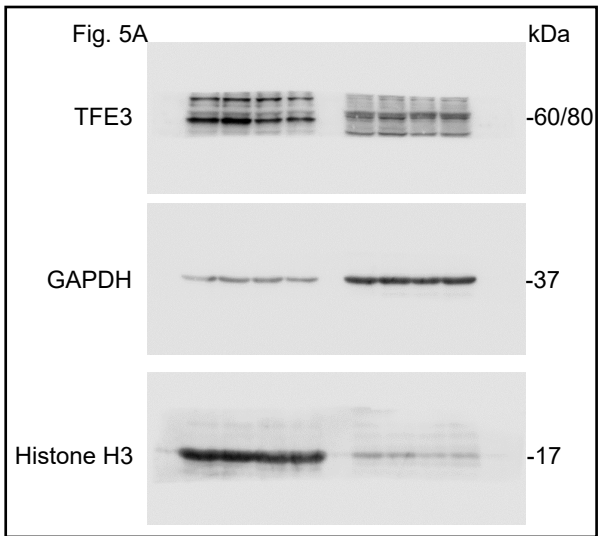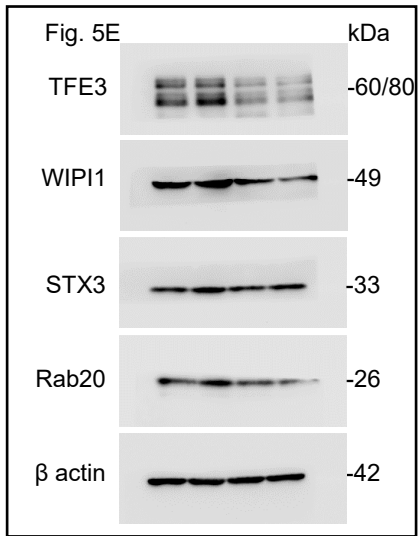

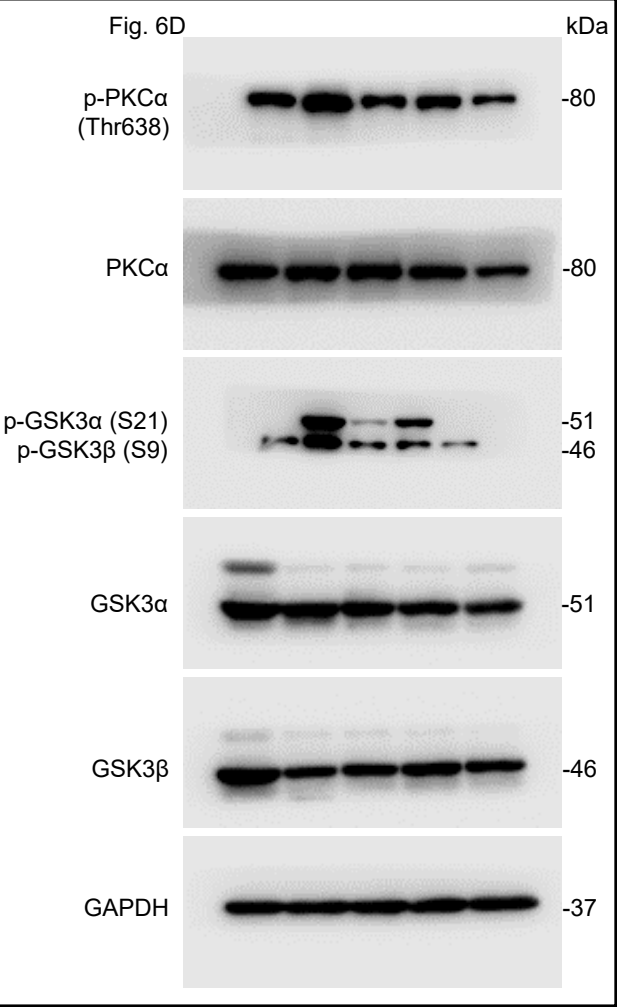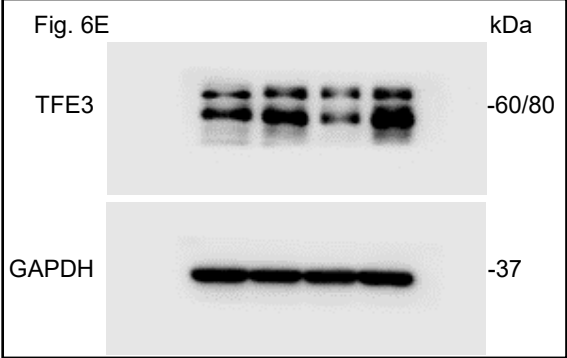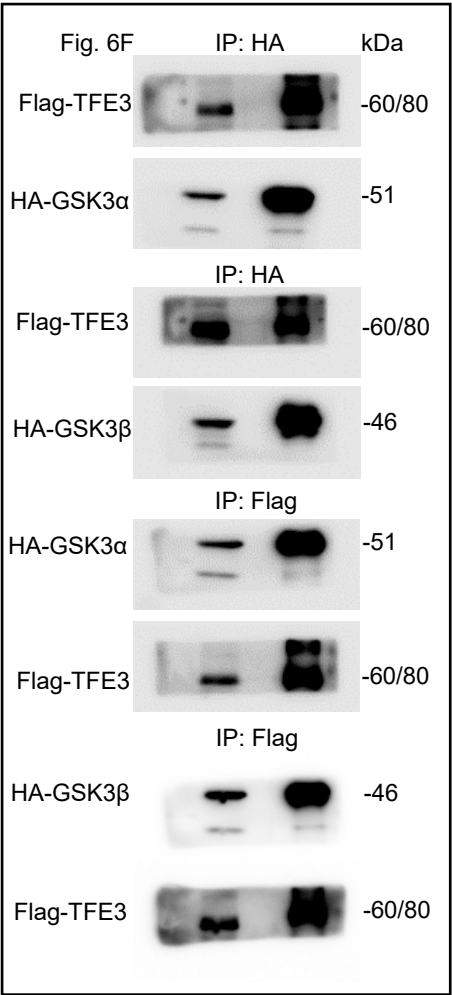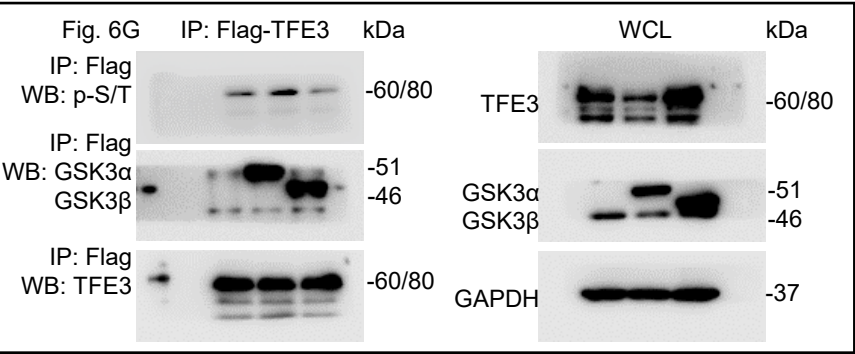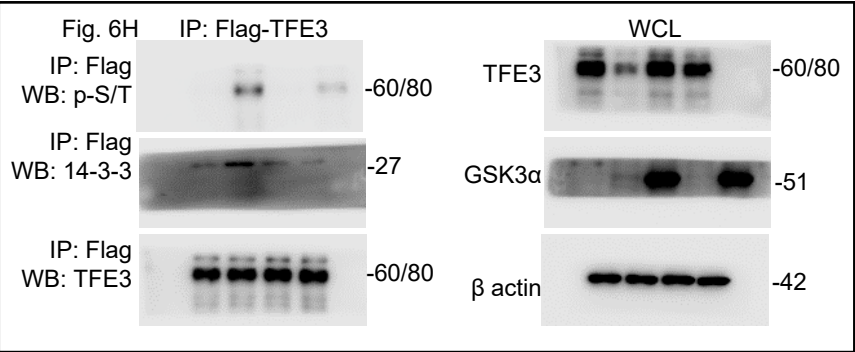

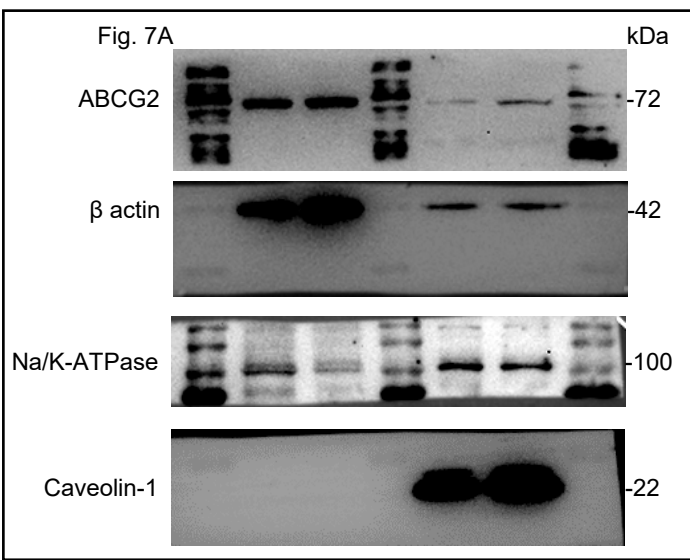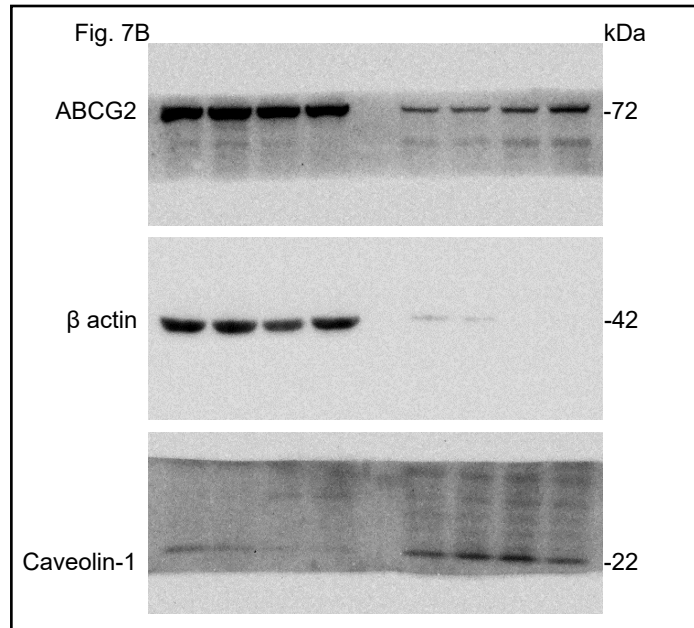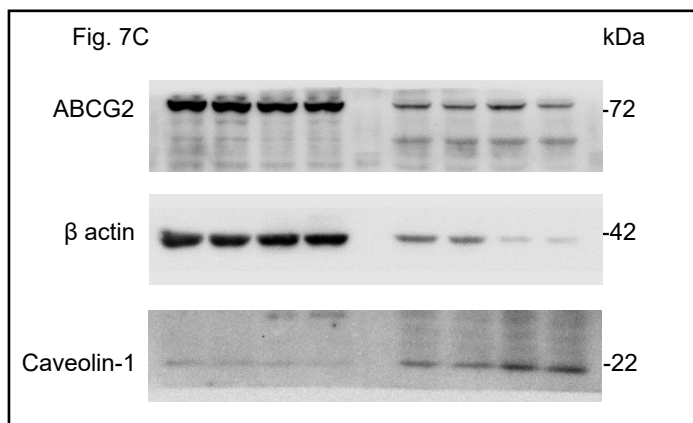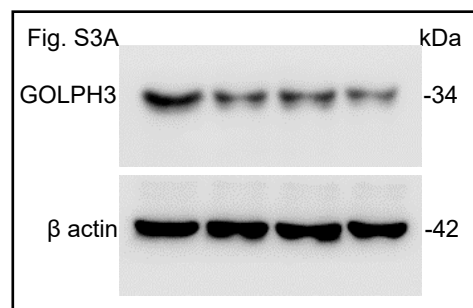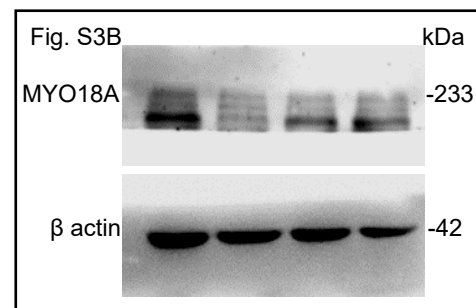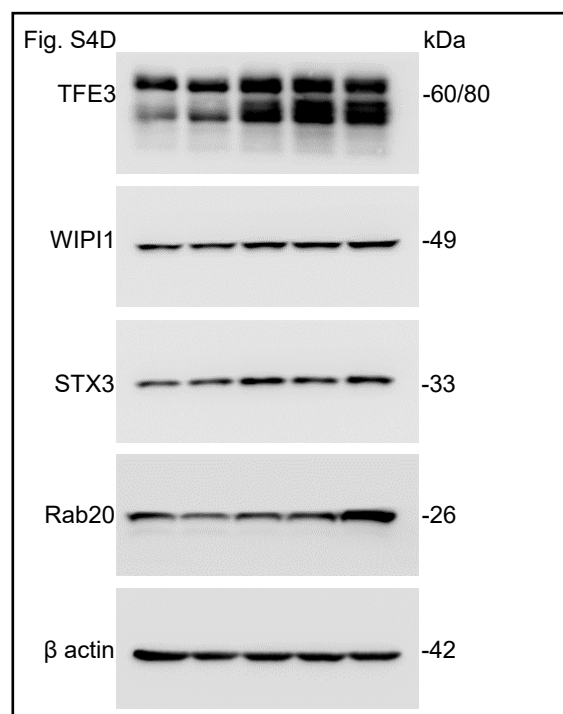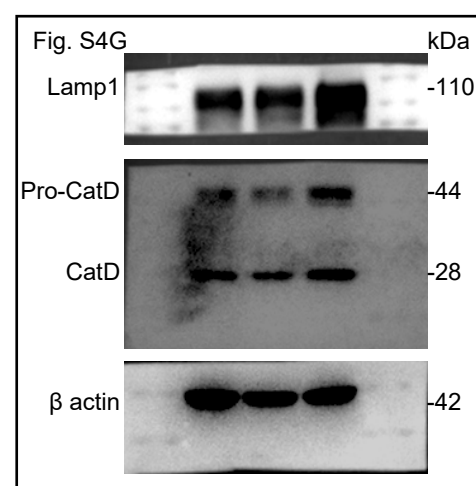

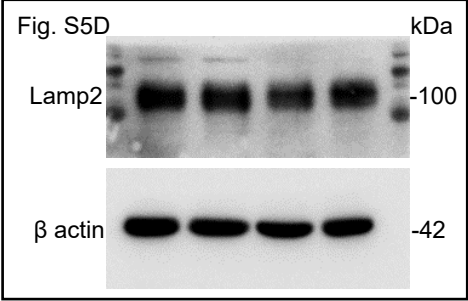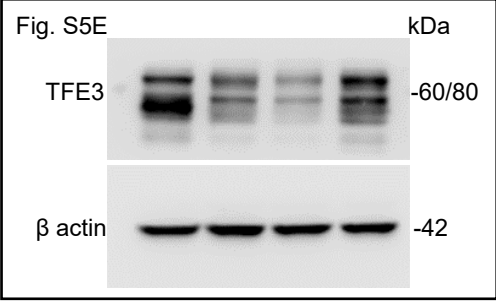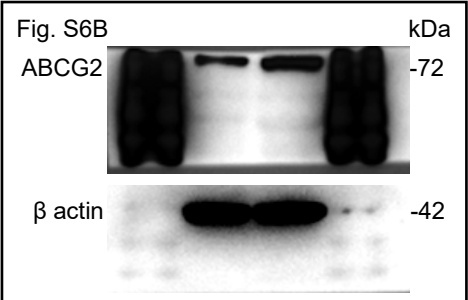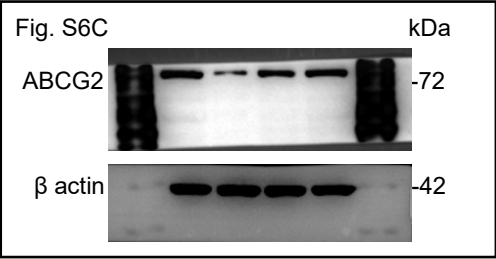

Supplement: Supplementary file 2 — Original Western Blots [file 41419_2024_6817_MOESM2_ESM.pdf]
